# Maternal obesity increases insulin resistance, low-grade inflammation and osteochondrosis lesions in foals and yearlings until 18 months of age

**DOI:** 10.1371/journal.pone.0190309

**Published:** 2018-01-26

**Authors:** M. Robles, E. Nouveau, C. Gautier, L. Mendoza, C. Dubois, M. Dahirel, B. Lagofun, M-C Aubrière, J-P Lejeune, I. Caudron, I. Guenon, C. Viguié, L. Wimel, H. Bouraima-Lelong, D. Serteyn, A. Couturier-Tarrade, P. Chavatte-Palmer

**Affiliations:** 1 UMR BDR, INRA, ENVA, Université Paris Saclay, Jouy en Josas, France; 2 Normandie Univ, UNICAEN, EA2608, OeReCa, USC-INRA, Caen, France; 3 Clinique Equine, Faculté de Médecine Vétérinaire, Université de Liège, Liège, Belgium; 4 IFCE, Station Expérimentale de la Valade, Chamberet, France; 5 INRA, UMR Toxalim, Research Center in Food Toxicology, Toulouse, France; Medical University of Vienna, AUSTRIA

## Abstract

**Introduction:**

Obesity is a growing concern in horses. The effects of maternal obesity on maternal metabolism and low-grade inflammation during pregnancy, as well as offspring growth, metabolism, low-grade inflammation, testicular maturation and osteochondrotic lesions until 18 months of age were investigated.

**Material and methods:**

Twenty-four mares were used and separated into two groups at insemination according to body condition score (BCS): Normal (N, n = 10, BCS ≤4) and Obese (O, n = 14, BCS ≥4.25). BCS and plasma glucose, insulin, triglyceride, urea, non-esterified fatty acid, serum amyloid A (SAA), leptin and adiponectin concentrations were monitored throughout gestation. At 300 days of gestation, a Frequently Sampled Intravenous Glucose Tolerance Test (FSIGT) was performed. After parturition, foals’ weight and size were monitored until 18 months of age with plasma SAA, leptin, adiponectin, triiodothyronine (T3), thyroxine (T4) and cortisol concentrations measured at regular intervals. At 6, 12 and 18 months of age, FSIGT and osteoarticular examinations were performed. Males were gelded at one year and expression of genes involved in testicular maturation analysed by RT-qPCR.

**Results:**

Throughout the experiment, maternal BCS was higher in O *versus* N mares. During gestation, plasma urea and adiponectin were decreased and SAA and leptin increased in O *versus* N mares. O mares were also more insulin resistant than N mares with a higher glucose effectiveness. Postnatally, there was no difference in offspring growth between groups. Nevertheless, plasma SAA concentrations were increased in O *versus* N foals until 6 months, with O foals being consistently more insulin resistant with a higher glucose effectiveness. At 12 months of age, O foals were significantly more affected by osteochondrosis than N foals. All other parameters were not different between groups.

**Conclusion:**

In conclusion, maternal obesity altered metabolism and increased low-grade inflammation in both dams and foals. The risk of developing osteochondrosis at 12 months of age was also higher in foals born to obese dams.

## Introduction

The influence of maternal environment on offspring long-term health has been demonstrated since the early nineties [1]. Many epidemiological studies in humans and experimental studies in animals have shown that maternal environment such as nutrition, metabolism or parity affects the *in utero* and post-natal development of the offspring, leading to increased susceptibility of developing non-communicable diseases at adulthood [2–4]. This concept is called the Developmental Origins of Health and Diseases (DOHaD) and has also been demonstrated in the horse [5].

In the equine species, the body condition can be measured by using the body condition score (BCS) method. The BCS is a subjective method based on overall appearance of the animal and palpation of selected areas [6–9]. There are currently two different scales used in the world: the 1–9 scale and the 1–5 scale [8,9]. The optimal BCS is set as 3/5 and 6/9 depending on the scale and a horse is considered fat with a BCS 4/5 and >8/9 in both systems [8,9]. Even if this method is largely used to estimate the fat content of a horse, there is yet no consensus on when is a horse overweight or obese based on these scales.

In the equine industry, obesity is a growing concern, especially since horses are increasingly being regarded as pets [10]. Surveys in Europe, North America, Australia and New-Zealand indicate that 2 to 72% of horses are considered as overweight and 1 to 19% as obese [11–26], with the lowest incidence observed in athletic horses involved in competition. Indeed, results vary depending on country, use of horses (leisure, competition) and season. Moreover, it has also been shown that horse owners consistently underestimate the body condition of their horses [12,14,17,21,23,24,27,28].

In feral or outdoor breeding conditions, the body condition of healthy horses varies depending on the availability of nutriments and thus with season, horses and ponies being fatter in summer than in winter [11,19,22,29–32]. Subsequently, it has been proposed that obesity in horses is defined as a stable obese condition (BCS>4) throughout the year, with no seasonal variation [11,33].

In horses, obesity has been linked to metabolic pathologies such as the equine metabolic syndrome, insulin resistance, alterations in adipose tissue endocrine function, low-grade inflammation and laminitis [34–42]. Moreover, decreased performances in races have been linked to higher body condition scores [43,44]. In terms of reproduction, winter anoestrus is reduced or absent in obese mares [45]. Foaling parameters do not appear to be affected by obesity [46,47]. So far, studies looking at effects of maternal obesity on foal development only focused on mares that were overfed during pregnancy and became obese at foaling [7,48]. To our knowledge, no data is available for mares that were already obese at the time of insemination.

The present study aims to investigate the effects of maternal obesity at the time of insemination on maternal metabolism and low-grade inflammation during gestation, and on foal growth, metabolism, low-grade inflammation, testicular maturation and osteoarticular lesions (focusing on osteochondrosis) until 18 months of age.

## Materials and methods

### Animals

#### Ethical statement

The animal studies were approved by the local animal care and use committee (“Comité des Utilisateurs de la Station Expérimentale de Chamberet”) and received ethical approval from the local ethics commitee (« Comité Régional d’Ethique pour l’Expérimentation Animale du Limousin ») under protocol number 5-2013-5.

#### Experimental design, management and feeding of mares and foals

**Fig 1:**
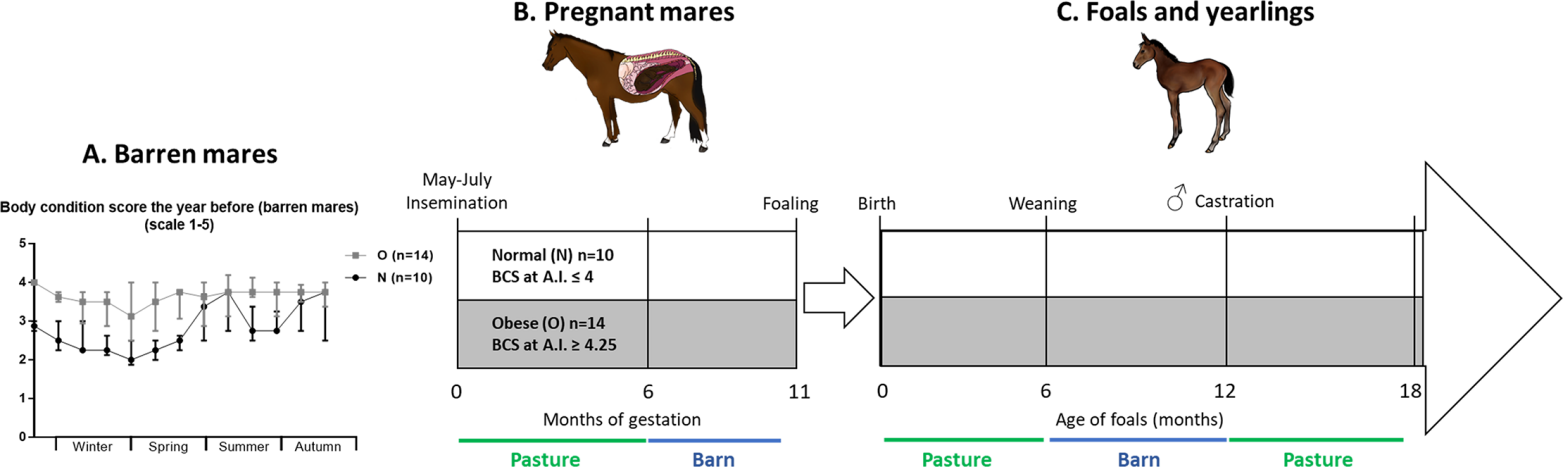
Experimental design. (A)The year before insemination, the body condition score of barren mares was monitored. Normal (N, n = 10) mares had a fluctuant body condition score relevant to the nutrient availability whereas Obese (O, n = 14) mares had a high and stable body condition during the year. (B)From insemination until 6 months of gestation, pregnant mares were pastured as one same herd in the same pasture. From the 6th month of gestation, they were housed in individual boxes and fed the same amount of energy, proteins, fibre, calcium and phosphorus according to body weight until foaling. (C)From 3 days until 6 months of age, foals were kept in pasture with their dam. They were weaned by abrupt separation at 6 months, housed in open barns and fed the same amount of feed, following the French recommendations for growing foals [49]. Colts were castrated at 12 months as a routine procedure. From 12 to 18 months of age, all yearlings pastured in the same pasture. A.I.: Artificial insemination, BCS: body condition score.

Experimental horses were raised in the “Institut Français du Cheval et de l’Equitation” experimental farm (Chamberet, France, 45°34'55.17''N, 1°43'16.29''E, 442 m). All animals were vaccinated and dewormed as for standard care. The experimental design is presented in Fig 1. Twenty-four multiparous barren mares (French Anglo-Arab and Selle Français breeds) were artificially inseminated with the same stallion (French Anglo-Arab breed) between the 5^th^ of May and the 5^th^ of July. To better being able to distinguish the effects of genetics in this study, a genealogy study has been performed (S1 Table) and showed that 7 stallions sired mares that were both in the N and the O group, 2 dams produced mares that were both in the N and the O group and 1 stallion was the grand sire of 3 mares that were distributed in both groups.

They were divided into two groups according to body condition score (BCS, 1–5 scale) at insemination. Ten mares (group Normal (N), mean age 7.9 years; range 6–12) had a BCS≤4 at insemination, with significant variation (mean 3.0, range 2–3.8) as barren mares during the previous year. Fourteen mares (group Obese (O), median age 8.6 years; range 6–11) had a BCS ≥4.25 at insemination that had been relatively stable during the previous year (mean 3.8, range 3.1–4), regardless of the nutrient availability (Fig 1A).

**Fig 2:**
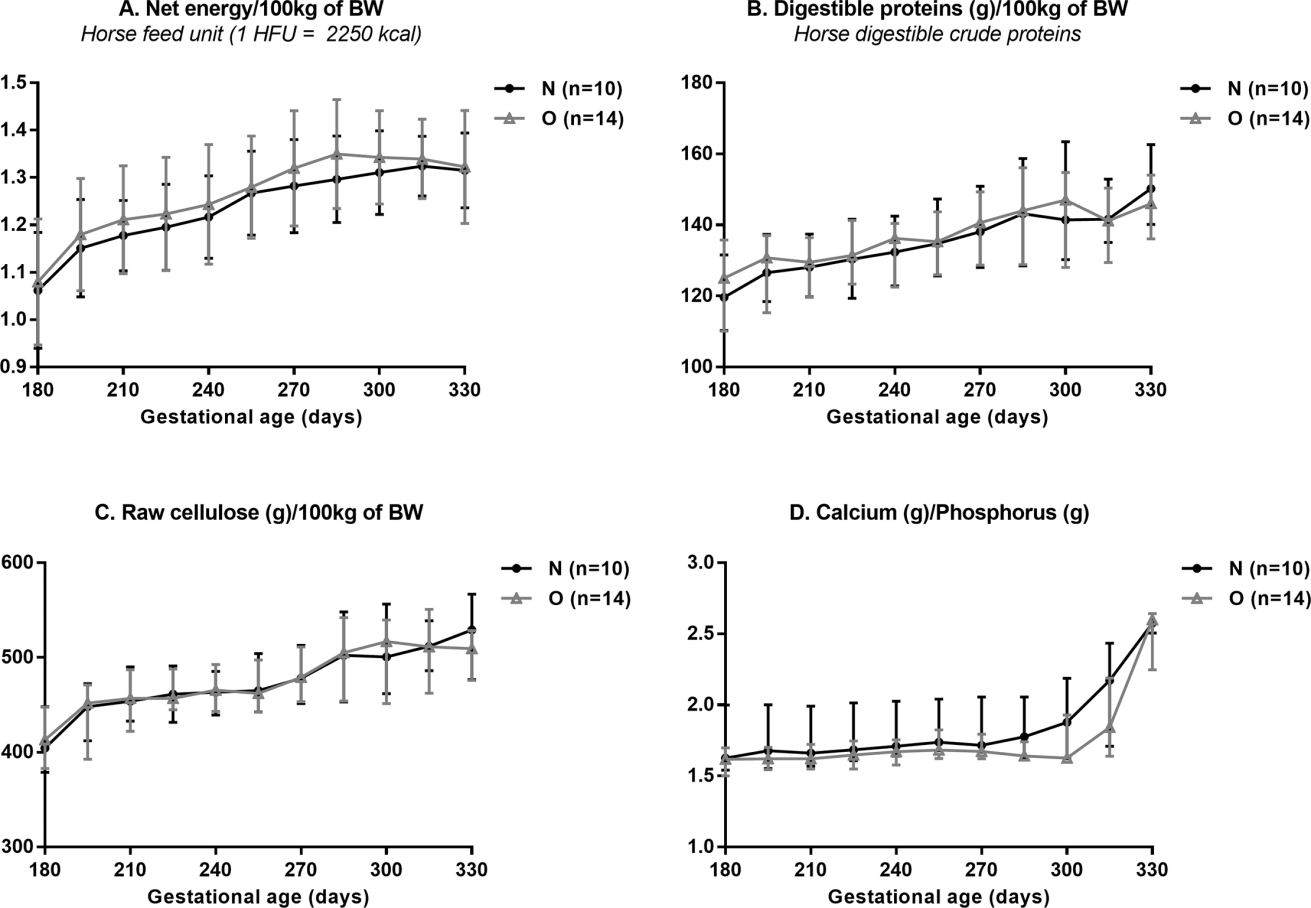
Daily nutritional information (median and IQR) of feed distributed to Normal (N) and Obese (O) broodmares from wintering at 180 days of gestation until foaling. There are no significant differences between groups. Net energy (A), horse digestible crude proteins (B), raw cellulose (C), and calcium to phosphorus ratio (D). BW: bodyweight.

From insemination to 6 months of gestation, pregnant mares were housed in one group in the same pastures with free access to water and mineral salts (Krouner Rumi, CTH, France). From the 6^th^ month of gestation, mares were housed in individual boxes and fed a diet based on hay, haylage and flattened barley with vitamins and minerals until foaling (Excel-Prima-S, Chauveau Nutrition, France), providing the same amount of energy, protein, fibres, calcium and phosphorus according to body weight (Fig 1B, Fig 2 and S2 Table).

Mares and foals returned to pasture 3 days after foaling (between the 30^th^ of April and the 17^th^ of June) and were kept in one herd in the same pasture until weaning at 6 months of age (between the 5^th^ and the 23^rd^ of November). In their first winter, foals were housed in open barns and fed hay and homemade pellets containing barley, soybean cake, molasses and vitamins and minerals (Excel-Prima-S, Chauveau Nutrition, France) twice a day using a collective feeder, in agreement with current recommendations for growing foals [49] (S3 Table). The animals were separated in three groups according to body weight in order to reduce the competition for food. Six months after weaning (April 25th, at 12 months of age), yearlings were returned to pasture and managed in one same herd with free access to water and a vitamin and minerals supplement (S4 Table) until 18 months of age (Fig 1C).

#### Body measurements and blood sampling of mares and foals

All measurements and sampling were performed without analgesia or anaesthesia.

Each month from insemination until weaning, mares were weighed and their BCS measured independently by two trained operators following the instructions of the Institut Français du Cheval et de l’Equitation (IFCE) based on the visual and manual analysis of 7 anatomical areas (neck, withers, shoulder, ribs, back, rump and tail head) [6]. Jugular vein blood samples were collected into EDTA-coated tubes monthly during pregnancy, in the morning (between 8:00 and 9:00 A.M) before the first meal of the day, to measure plasma glucose, insulin, triglyceride (TG), non-esterified fatty acid (NEFA), urea, leptin, adiponectin and serum amyloid A (SAA) concentrations.

All mares foaled naturally and foaling was supervised. At birth before suckling and at 1, 2, 3, 4, 5, 6, 12 and 18 months, foals and yearlings were weighed and withers’ height and chest width measured. The BCS of yearlings was measured at 330, 450, 480 and 600 days of age by two trained, independent operators. Jugular vein plasma and serum samples were collected into EDTA-coated or dry tubes at birth before suckling to measure plasma leptin, adiponectin, SAA concentration and cortisol serum concentration. Jugular vein plasma samples were collected into EDTA-coated tubes at 1, 3, 6 and 12 months in the morning (before the first meal during wintering), to measure plasma leptin, adiponectin and SAA concentrations. At 4 months, foals were catheterized at the jugular vein. After 30 minutes, 3 blood samples were collected into tubes coated with a coagulation activator at 30 min intervals to measure serum cortisol concentration.

### Evaluation of glucose metabolism in mares, foals and yearlings

#### Modified frequently sampled intravenous glucose tolerance test (FSIGT)

Modified FSIGT were performed at 300 days of gestation in mares and at ages 6, 12 and 18 months in foals and yearlings. The FSIGT is a method that enables the simultaneous evaluation of insulin sensitivity and glucose tolerance [50]. This method does not require overnight fasting so animals had free access to hay and water during the test. Both jugular veins were catheterized (Introcan, BBraun, Germany) 30min before the beginning of the test. One catheter was used for infusion and the other one for sampling. Glucose (0.30g/kg of body weight) was injected over 2 min (age 6 months), 4 min (age 12 months) and 5 min (mares at 300 days of gestation and yearlings at age 18 months) and 10 ml samples were collected into EDTA-coated tubes at -5 min and 5, 7, 13 and 19 min after the glucose injection started. At 20 min, a diluted solution (2.51mUI/L) of insulin (Umuline 100mUI/L, Eli Lilly, USA) was injected over 1 min at the concentration of 15mUI/kg of body weight. Blood was collected at 5, 15, 25, 40, 70, 100, 130 and 160 min after insulin injection. Whole blood glucose concentration was immediately analysed using a glucometer (Freestyle optium, Abbott, USA). Samples were kept on ice until centrifugation (0–2 h). Samples were stored at -20°C until use.

#### Insulin assays

Plasma insulin concentrations were measured using an AlphaLISA human insulin immunoassay kit (PerkinElmer, USA) as previously described and validated [51]. The minimum level of detection was 5.3 mUI/L. Intra- and inter-assay coefficients of variation were 6% and 7%, respectively.

#### Calculation of glucose homeostasis parameters from modified FSIGT assays

Glucose effectiveness (Sg), acute insulin response to glucose (AIRg), insulin sensitivity (SI) and the disposition index (DI) were calculated using the Bergman’s minimal model [50,52,53] on the MinMod Millennium software (Ver 6.02, MINMOD Inc., 2001) [54]. A schematic representation of the Bergman’s minimal model has been described previously [51]. The goodness of fit r^2^ of models for all mares was 91.5% (range 74.6–98.22) and all the parameters were estimated with a fractional standard deviation (FSD) <0.5.

Briefly, glucose and insulin are produced and released by the liver and the pancreas respectively in a basal state (basal plasma glucose concentration, Gb; basal plasma insulin concentration, Ib). After a meal, or an intra-venous injection of glucose, plasma glucose concentrations increase. From the blood compartment, the glucose will enter the peripheral tissues by two types of glucose transporters, leading to the measurement of different indices:

By insulin independent glucose transporters. The glucose efficiency (Sg) index is the capacity of the glucose to mediate its own disposal independently of insulin and to suppress endogenous glucose production.

By insulin dependent glucose transporters. The elevated level of plasma glucose induces the production of insulin by the pancreas. The acute insulin response (AIRg) index represents the production of insulin by the pancreas during the first 10 minutes after glucose injection and reflects β-cells responsiveness. Insulin is transferred into the interstitial space (P3, insulin introduction rate) from the bloodstream and reaches the peripheral tissues to mediate glucose disposal by insulin dependent glucose transporters (X, insulin action). With time, insulin action declines at a rate calculated by the P2 proxy. The Insulin sensitivity (SI) index is then calculated as P2/P3.

Finally, the disposition index (DI) (calculated as the product of SI and AIRg) is used to describe the whole-body insulin sensitivity.

### Biochemical analyses

#### Plasma TG, NEFA and urea analyses

Plasma TG, NEFA and urea concentrations were measured in mares in duplicate by an enzymatic-colorimetric method with a Cobas Mira-analyzer, using commercial kits (TG: Provet triglycerides SL, Kitvia, France; NEFA: NEFA-HR kit, Wako Chemicals GmbH, Germany; urea: Provet urea SL,Kitvia, France) and following the manufacturers’ instructions. For TG assay, intra- and inter-assay coefficients of variation were 4.6% and 7.2%, respectively. For NEFA assay, intra- and inter-assay coefficients of variation were 2.3% and 2.7%, respectively. For urea assay, intra- and inter-assay coefficients of variation were 3.0% and 6.9%, respectively.

#### Plasma leptin and adiponectin analysis

Plasma leptin and adiponectin concentrations were measured in mares, foals and yearlings in duplicate using an alphaLISA human leptin and adiponectin immunoassay kit (PerkinElmer, USA) following the manufacturer’s instructions, as described previously for insulin [51].

For leptin, the minimum level of detection was 0.26ng/mL. Intra- and inter-assay coefficients of variation were 8% and 8.3%, respectively. For adiponectin, the minimum level of detection was 0.019ng/mL. Intra- and inter-assay coefficients of variation were 8% and 8.9%, respectively. The assays were validated by dilutional parallelism between standard curve and endogenous leptin or adiponectin and expected values against obtained values (S1 and S2 Figs).

#### Plasma SAA analysis

Plasma SAA concentrations were measured in mares, foals and yearlings in duplicate with a commercial multi-species ELISA kit following the manufacturer’s instructions (Phase^TM^ range SAA kit, Tridelta Development Ltd, Ireland). This commercial kit has been validated for use in horses by the manufacturer, intra- and inter-assay coefficients of variation were 4.6% and 8.5%, respectively.

#### Serum cortisol analysis

Serum cortisol concentrations were measured in foals at birth before suckling and at 4 months of age in duplicate with a commercial human RIA kit following the manufacturer’s instructions (CORT-CT2, CISbio International, France). The 3 plasma samples collected at 4 months of age were averaged as there was no difference for cortisol concentration between them. Intra- and inter-assay coefficients of variation were 3.4% and 6.0%, respectively.

#### Thyroid hormones analysis

Total T3 and total T4 were assayed using MP Biomedicals (Solon, USA) radiommunoassay kits in aliquots of 25 or 10 μl and 50 to 200μl, for T4 and T3, respectively. These assays were validated for horse serum by measuring T3 and T4 in horse plasma spiked with four different concentrations levels: 0, 20, 50 and 100ng/ml and 0, 1, 2.5 and 5ng/ml for T4 and T3, respectively. For T4 assay, mean inter- and intra-assay CVs were 16.0 and 5.7%, respectively and mean sensitivity according to expected concentrations in spiked samples was -12.4%. For T3, mean inter- and intra-assay CVs were 9.4 and 13.4%, respectively and mean sensitivity according to expected concentrations in spiked samples was -14.5%.

### Testis

#### Tissue sampling and treatment

Testes from prepubertal stallions (group N: n = 7, mean age 304 days, range 265–319 and group O: n = 7, mean age 306 days, range 281–314) were collected during routine castration. Samples were snap frozen on dry ice and stored at -80°C.

#### Quantitative real time PCR

Total mRNA was extracted with Tri-Reagent kit (Sigma-Aldrich, France) according to the manufacturer’s instructions. Reverse transcription and PCR reactions were performed as previously described [51]. The relative gene expression was normalized with *GAPDH* RNA expression.

The genes analysed were markers of testicular maturity (*Cyp19*, *Connexin 43*, *AR*, *STAR* [55–57]), markers of immaturity (*AMH* [58]) and genes involved in the establishment of the blood-testis barrier (*Occludin*, *N Cadherin*). Primer sequences are presented in Table 1.

**Table 1:** Primers sequences used for amplification of equine products in RT-qPCR assays in testicles of prepubertal stallions.

Gene	GenBank accession number	Primer	Size of PCR product (pb)
***Anti-müllerian hormone (AMH)***	JF330269,1	Forward: 5'—CCATCTGGAGGAGCCAAC—3'	80
		Reverse: 3'—CCCGTGACAGTGACCTCAG—5'	
***Androgen Receptor (AR)***	NM_001163891,1	Forward: 5'—AGTACTCCTGGATGGGGCTT—3'	132
		Reverse: 3'—TGTACATCCGGGACTTGTGC—5'	
***Connexin 43 (Cx43)***	XM_005596901,1	Forward: 5'—CTCTTCACCAACCGCTCCT—3'	68
		Reverse: 3'—CTGTCTCCGGTAACCAGCTT—5'	
Cyp 19	NM_001081805,1	Forward: 5' -AAAGCCACCCGGTTCCTAAC- 3'	119
		Reverse: 3' -CCTTGATCTCAGGCGAAGCA- 5'	
***N Cadherin***	XM_001495782,3	Forward: 5' -GCCATTCAGACTGACCCGAA- 3'	100
		Reverse: 3'—CTGCAGCGACAGTAAGGACA- 5'	
***Occludin***	XM_001504044,3	Forward: 5' -AAGCTTCCATGTCAGTGCCTT- 3'	84
		Reverse: 3' -TTGCTTGGTGCGTAATGATTGG—5'	
***Steroidogenic Acute Regulatory Protein (STAR)***	NM_001081800,1	Forward: 5'—TGGCCTATATCCAGCAGGGA- 3'	142
		Reverse: 3' -AATACCTTGCCCACGTCTGG- 5'	

### Radiographic evaluation of osteoarticular status in yearlings

Radiographic examination at 6, 12 and 18 months of age was performed based on 8 X-rays of the hock, stifle and fetlock as recommended by Denoix and collaborators [51,59]. Foals and yearlings were sedated using butorphanol (Dolorex®, 0.02mg/kg IV, Intervet, USA) combined with romifidine (Sedivet®, 0.06mg/kg IV, Intervet, USA) when needed. OC was diagnosed blindly by two experienced examinators according to the presence of lesions that were previously described [60–62]. Yearlings were classified as OC-positive according to the presence of one or more OC lesions identified by both examiners.

### Statistical analysis

Results are expressed as median [quartile 1 –quartile 3] and presented as curves (median and interquartile range IQR) or boxplots (minimum to maximum). Statistical analyses were performed using the R software (http://www.r-project.org, version 3.4.0.).

The longitudinal data (mare weight and BCS, plasma concentrations in mares and foals and foals’ metabolic tests) were analysed using a type III ANOVA (*car* package [63]) on a mixed linear model (*nlme* package[64]) taking into account group (N or O), time and the interaction group:time as fixed effects and the individual as random effect. Age of the mare and sex of the foal were included in the model when statistically significant. When time and/or group:time interaction were statistically significant, a post-hoc Tukey test was applied using the *lsmeans* package [65]

Foal and yearling measurements were analysed using a mixed quadratic model considering the group (N or O), the age of the foal (from birth to 18 months of age) and the interaction group:age of the foal as fixed effects and the foal as random effect. When age and/or group:age interaction were statistically significant, a post-hoc Tukey test was applied using the *lsmeans* package. Age of the mare and sex of the foal were not statistically significant at any time and were subsequently not included in the model.

Metabolic tests performed in mares in late gestation were analysed using a type III ANOVA (*car* package) on a fixed linear model considering the group (N or O) and age of the mare.

Testicular data were analysed using a Principal Component Analysis of the FactoMineR package [66]. The age and weight of the foal at gelding did not affect any of the results and were therefore not included in the final analysis.

Radiographic results were analysed using a chi-squared test.

Effects were considered significant when p-values<0.05.

## Results

### Effect of obesity in mares

#### Bodyweight and BCS in mares

**Fig 3:**
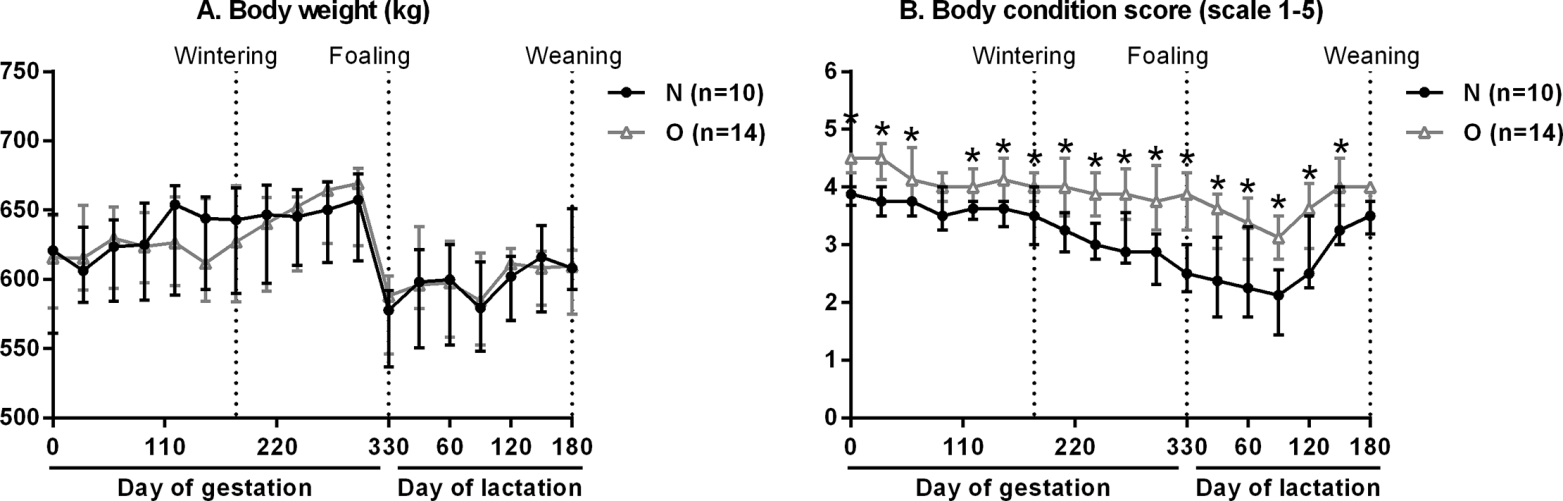
**Bodyweight (A) and body condition score (B) of mares (median and IQR) throughout pregnancy and lactation.** For each time point, asterisks indicate a significant difference (p<0.05).

Gestation and lactation bodyweight and BCS of mares are shown in Fig 3. There was no difference between groups for maternal weight from insemination until weaning. O mares, however, gained weight from wintering at 180 days of gestation until 300 days of gestation (mean 35.1kg, range 0.8–59.9kg, p<0.0001) but N mares did not.

O mares had a higher BCS than N mares from insemination until the month before weaning of the foals (O mean 3.9, range 2.0–5.0, N mean 3.2, range 1.3–4.5, p<0.0001). N mares lost body condition from 180 days of gestation to foaling (mean 0.78, range 0–1.5, p<0.0001) whereas O mares showed only small significant changes of BCS during this period. N mares gained body condition between foaling and weaning of foals at 180 days of age (mean 0.78, range 0–1.25, p<0.0001) but O mares did not, so that altogether, the body condition of O mares did not vary very much in contrast to that of N mares.

#### Glucose homeostasis in mares

**Fig 4:**
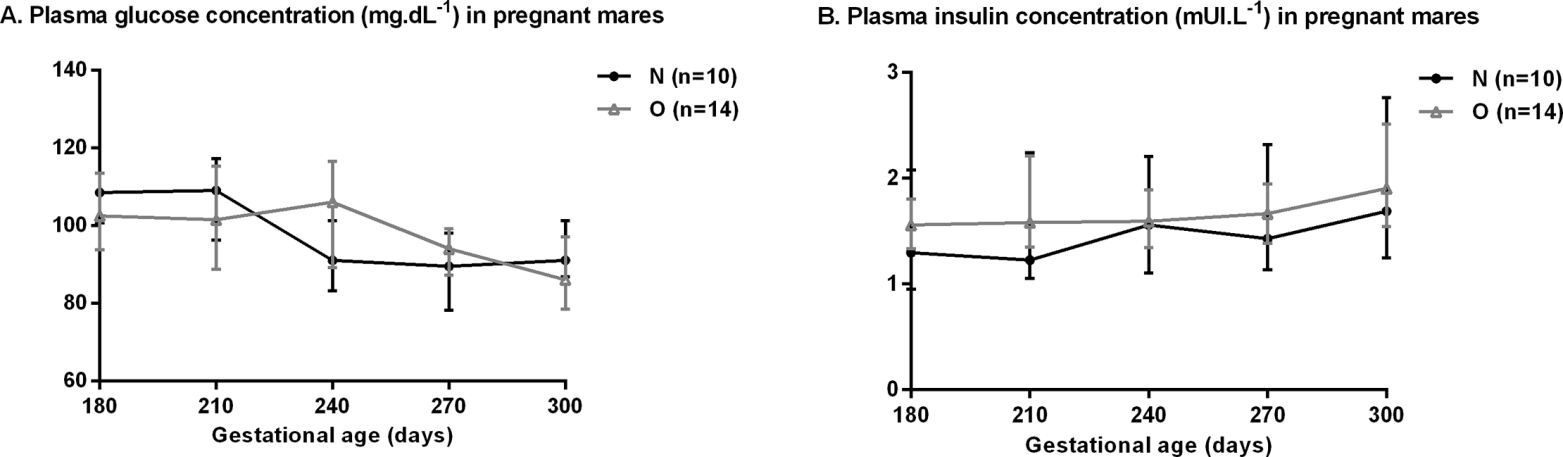
Basal plasma glucose (A) and insulin (B) concentration in pregnant Normal (n = 10) and Obese (n = 14) mares between 180 and 300 days of gestation.

Data from glucose metabolism analyses are presented in Fig 4 and Table 2.

**Table 2:** Indexes calculated with the Bergman’s minimal model (median and IQR): Acute insulin response to glucose (AIRg), insulin sensitivity (IS), disposition index (DI) and glucose effectiveness (Sg), basal plasma glucose (GB) and basal plasma insulin (IB) in Normal (n = 10) and Obese (n = 14) mares at 300 days of gestation.

	Normal (n = 10)	Obese (n = 14)	P value
AIRg mUI.min.L^-1^	72.3 [43.3–112.7]	101.6 [64.0–205.1]	NS
**SI x10**^**-4**^ **L.mUI**^**-1**^**.min**^**-1**^	**1.9 [1.0–2.4]**	**0.5 [0.4–0.9]**	**<0.01**
DI x10^-2^	123.2 [93.1–182.7]	83.7 [32.3–155.3]	NS
**Sg x10**^**-2**^**.min**^**-1**^	**0.008 [0.004–0.009]**	**0.011 [0.010–0.013]**	**0.02**
GB mg.dL^-1^	104 [95–116]	103 [91–118]	NS
IB mUI.L^-1^	21.1 [15.3–31.9]	29.8 [20.1–41.1]	NS

There was no difference for basal plasma glucose and insulin concentrations between groups from 180 to 300 days of gestation. Plasma glucose concentrations decreased with pregnancy (p<0.0001) while plasma insulin concentration increased (p<0.001). There was no difference between groups for acute pancreatic response (AIRg), disposition index (DI), basal glucose and insulin concentrations.

At 300 days of gestation, however, O mares were more insulin resistant than N mares (p = 0.001) and had a higher Sg (glucose effectiveness, p = 0.02).

#### Plasma biochemistry in mares

**Fig 5:**
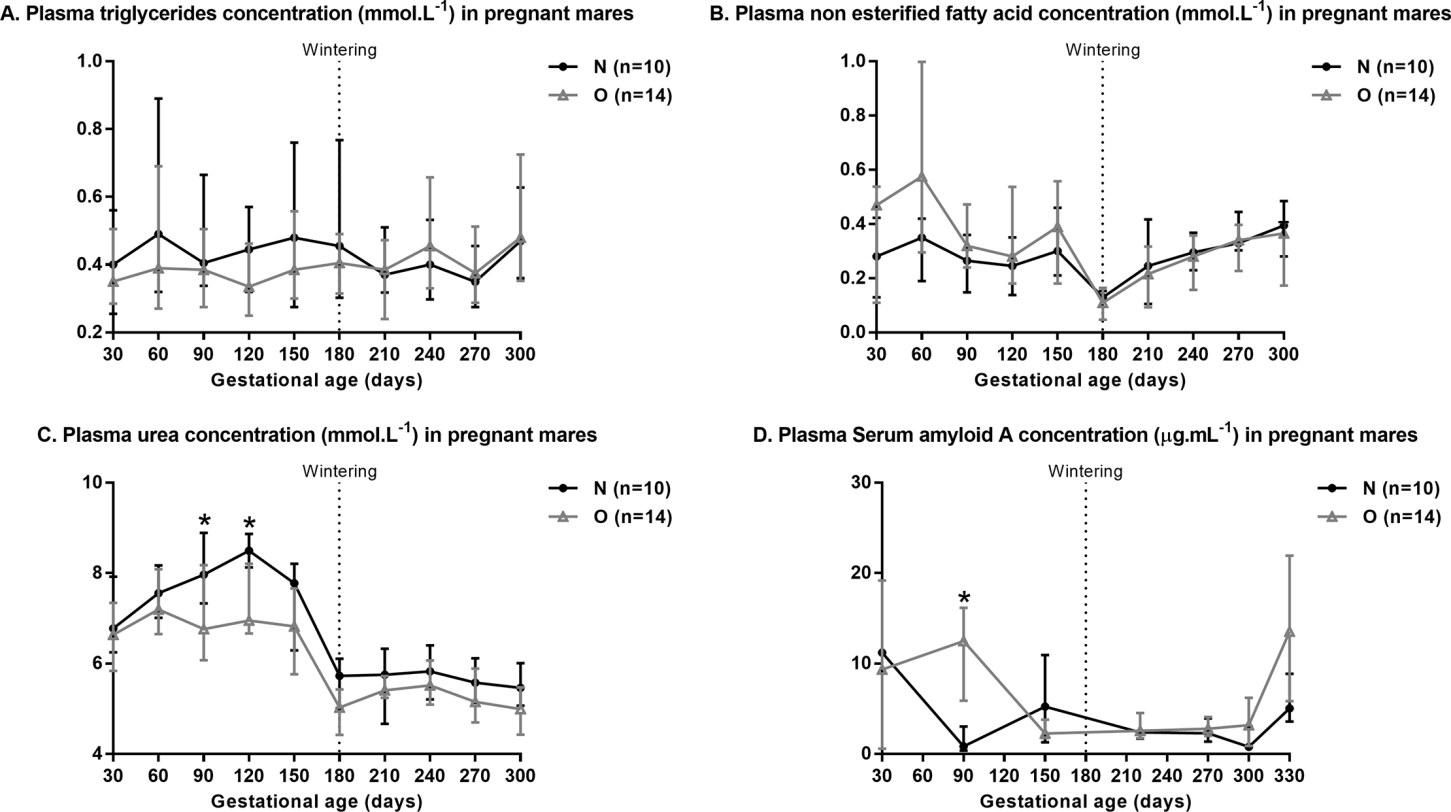
**Plasma triglycerides (A), non-esterified fatty acid (B), urea (C) and Serum amyloid A (D) concentration in pregnant Normal (n = 10) and Obese (n = 14) mares between 30 and 300 days of gestation or foaling.** For each time point, asterisks indicate a significant difference between groups (p<0.05).

Plasma biochemistry results are shown in Fig 5.

There was no difference between groups for plasma TG and NEFA concentrations between 30 and 300 days of gestation (Fig 5A and 5B). Plasma NEFA concentrations, however, increased from wintering at 180 days of gestation to 300 days of gestation (p = 0.0001) in both groups without any effect of the group:time interaction.

Plasma urea concentrations tended to be decreased in O compared to N mares when comparing all measurements throughout gestation (p = 0.07) (Fig 5C). Indeed, plasma urea concentrations were reduced in O compared to N mares at 90 (p<0.01) and 120 (p = 0.01) days of gestation. Plasma urea concentration decreased between 120 and 180 days of gestation (p<0.0001) in both groups of mares without any effect of the group:time interaction.

Altogether, plasma SAA concentrations were higher in O compared to N mares (p = 0.04) from 30 days of gestation to foaling. Plasma SAA concentrations were higher in O mares at 90 days (p<0.01). Moreover, plasma SAA concentrations increased in O mares between 300 days of gestation and foaling (p = 0.03) while it stayed stable in N mares during this period.

#### Plasma endocrinology in mares

**Fig 6:**
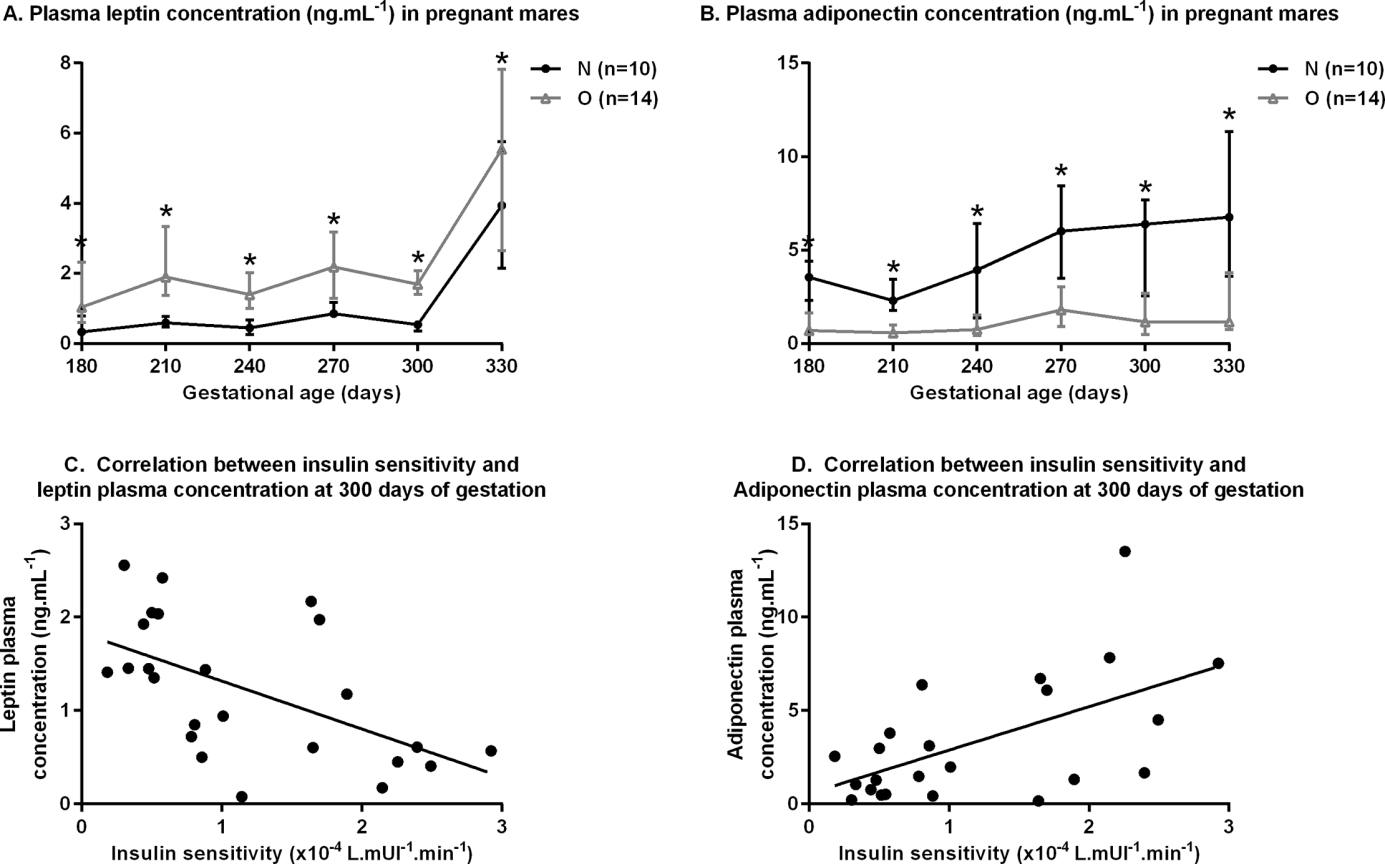
**Plasma leptin (A) and adiponectin (B) concentrations in pregnant Normal (n = 10) and Obese (n = 14) mares from 180 days of gestation to foaling and correlations between insulin sensitivity calculated using the Bergman’s minimal model and plasma leptin concentrations (C) or plasma adiponectin concentrations (D) at 300 days of gestation.** For each time point, asterisks indicate a significant difference between groups (p<0.05) and “T” indicate a tendency for a difference between groups (p<0.10).

Plasma concentrations of leptin and adiponectin are shown in Fig 6.

From 180 days of gestation to foaling, O mares had higher plasma leptin and lower plasma adiponectin concentrations than N mares (p<0.0001, p<0.0001, respectively). There was no effect of the group:time interaction for plasma leptin concentration but the plasma adiponectin concentration increased during gestation in N mares (p = 0.01) while it stayed stable in O mares.

Moreover, there was a negative linear correlation between plasma leptin concentration and SI (p<0.01, adjusted r^2^ = 0.29) and a positive linear correlation between plasma adiponectin concentration and SI (p<0.01, adjusted r^2^ = 0.31) at 300 days of gestation.

### Effect of maternal obesity in foals and yearlings

#### Body measurement in foals and yearlings

**Fig 7:**
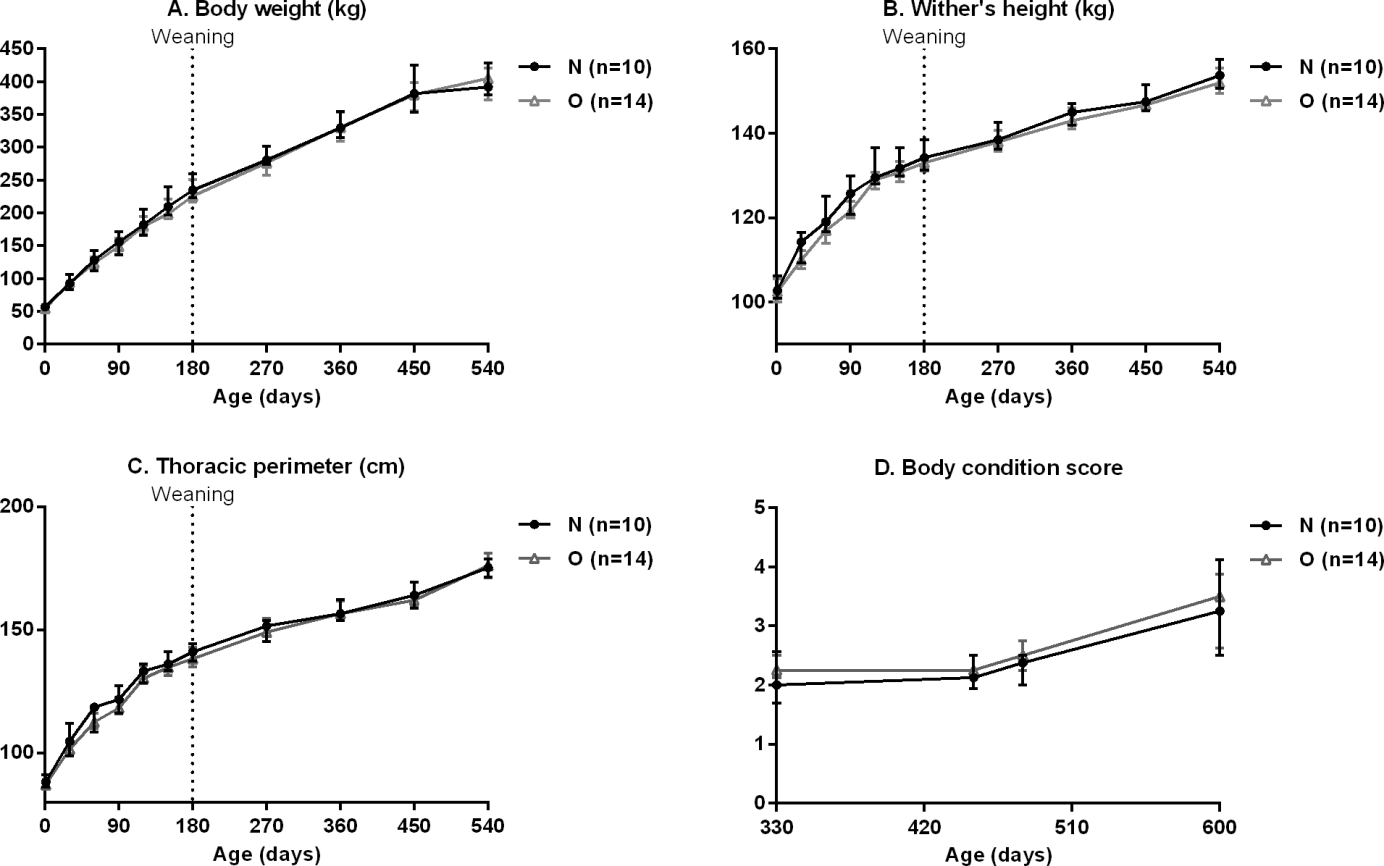
**Body weight (A), wither’s height (B), chest width (C) and body condition score (D) in Normal (n = 10) and Obese (n = 14) growing foals and yearlings.** There was no difference between groups.

Growth of foals is shown in Fig 7.

There was no difference in growth between groups from birth to 18 months of age. There was no difference in BCS from 330 to 600 days of age. There was no effect of group:time interaction for body mensurations in foals and yearlings.

#### Glucose homeostasis in foals

Data from glucose metabolism analyses are shown in Table 3.

**Table 3:** Indexes calculated with the Bergman’s minimal model (median and IQR): Acute insulin response to glucose (AIRg), insulin sensitivity (SI), disposition index (DI) and glucose effectiveness (Sg), basal plasma glucose (GB) and basal plasma insulin (IB) in Normal (n = 10) and Obese (n = 14) foals and yearlings at 6, 12 and 18 months of age.

	6 months		12 months		18 months		Time p value
	Normal (n = 10)	Obese (n = 14)	Normal (n = 10)	Obese (n = 14)	Normal (n = 10)	Obese (n = 14)	
AIRg mUI.min.L^-1^	14.9 [11.1–37.6]	30.9 [8.1–39.2]	68.1 [47.9–84.9]	80.7 [70.7–89.6]	80.2 [31.3–84.5]	65.8 [54.4–111.1]	<0.0001
SI x10^-4^ L.mUI^-1^.min^-1^	**17.2 [9.5–26.9]**^**a**^	**4.5 [3.4–7.9]**^**b**^	1.9 [1.5–2.3]	2.4 [1.4–2.5]	**5.6 [1.5–7.9]**^**a**^	**0.9 [0.6–2.6]**^**b**^	<0.0001
DI x10^-2^	290.9 [145.1–400.9]	115.7 [37.9–330.8]	122.9 [89.9–160.6]	119.6 [107.1–197.6]	246.7 [165.5–324.6]	67.6 [42.9–173.1]	NS
Sg x10^-2^.min^-1^	0.009 [0.003–0.013]	0.009 [0.007–0.014]	0.010 [0.006–0.012]	0.008 [0.005–0.011]	**0.022 [0.016–0.030]**^**a**^	**0.010 [0.006–0.015]**^**b**^	<0.0001
GB mg.dL^-1^	87.5 [85.5–99.5]	93.8 [87.1–100.4]	117.8 [111.8–125.3]	123.0 [107.5–127.5]	111.3 [96.5–120.5]	123.5 [105.9–146.4]	<0.0001
IB mUI.L^-1^	8.7 [8.2–12.1]	9.6 [9.4–10.5]	5.9 [5.4–9.6]	8.0 [6.3–11.5]	7.6 [4.0–10.0]	9.4 [5.0–12.0]	NS

Different suffixes indicate a significant difference between groups (p<0.05).

At 6 and 18 months of age, O foals had a reduced SI compared to N foals (p<0.0001, p = 0.03, respectively). Sg was also reduced in O foals at 18 months of age (p = 0.002).

AIRg, Sg and basal glucose increased with age (p<0.0001, p<0.0001, p<0.0001, respectively) whereas SI decreased with age (p<0.0001). SI decreased between 6 and 12 months of age in the N group (p<0.001) but not in the O group. There was no other effect of interaction group:time in AIRg, DI, Sg, IB and GB.

#### Chronic low-grade inflammation in foals

**Fig 8:**
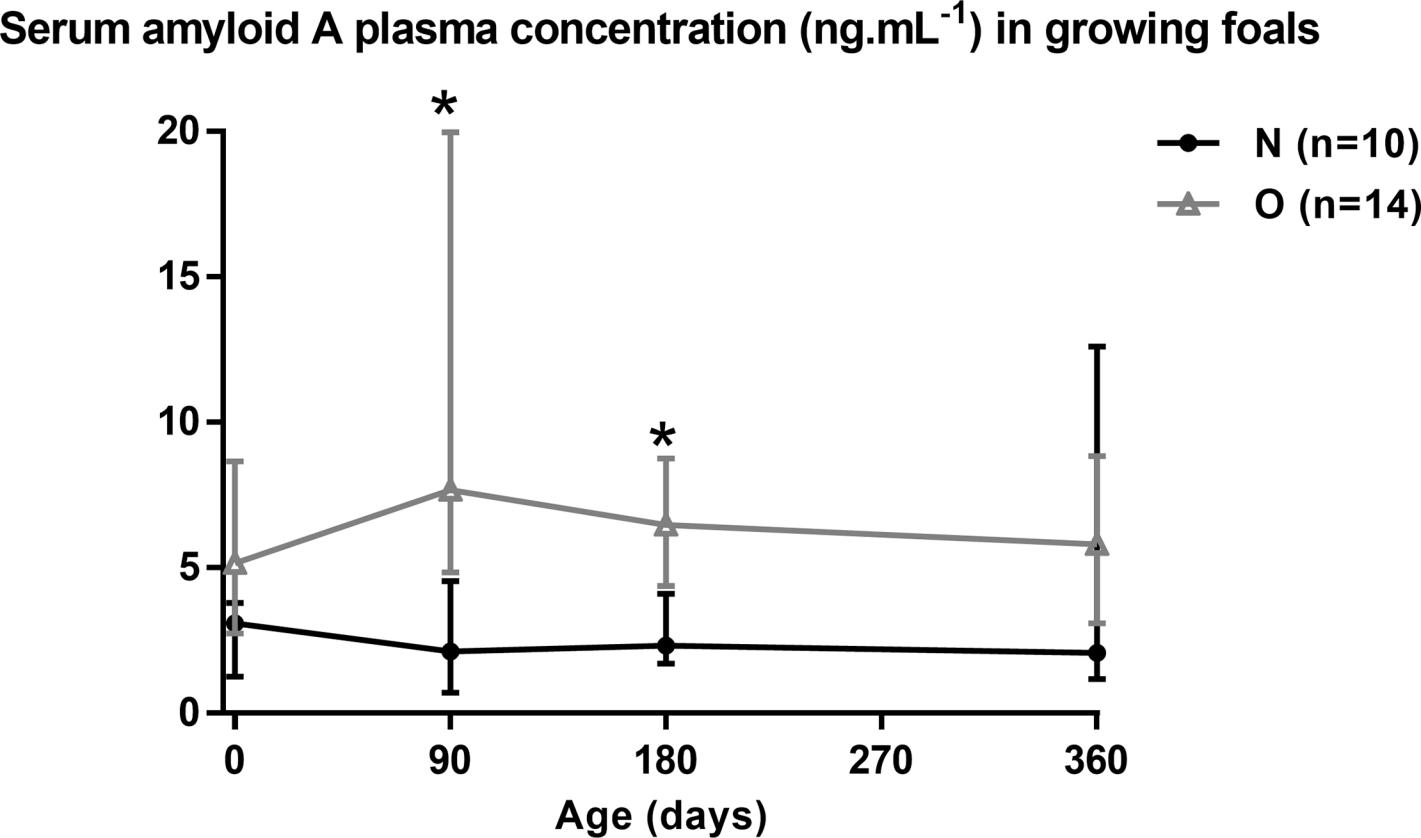
Plasma Serum amyloid A concentrations in Normal (n = 10) and Obese (n = 14) foals and yearling from birth to 12 months of age. For each time point, asterisks indicate a significant difference between groups (p<0.05).

Chronic low-grade inflammation as evaluated by plasma SAA concentrations are shown in Fig 8.

Altogether, plasma SAA concentrations were higher in O compared to N foals throughout growth (p = 0.048). Plasma SAA concentrations were significantly higher in O foals at 3 (p<0.01) and 6 (p = 0.01) months of age compared to N foals. There was no effect of group:time interaction for the plasma SAA concentrations.

#### Foal endocrinology

**Fig 9:**
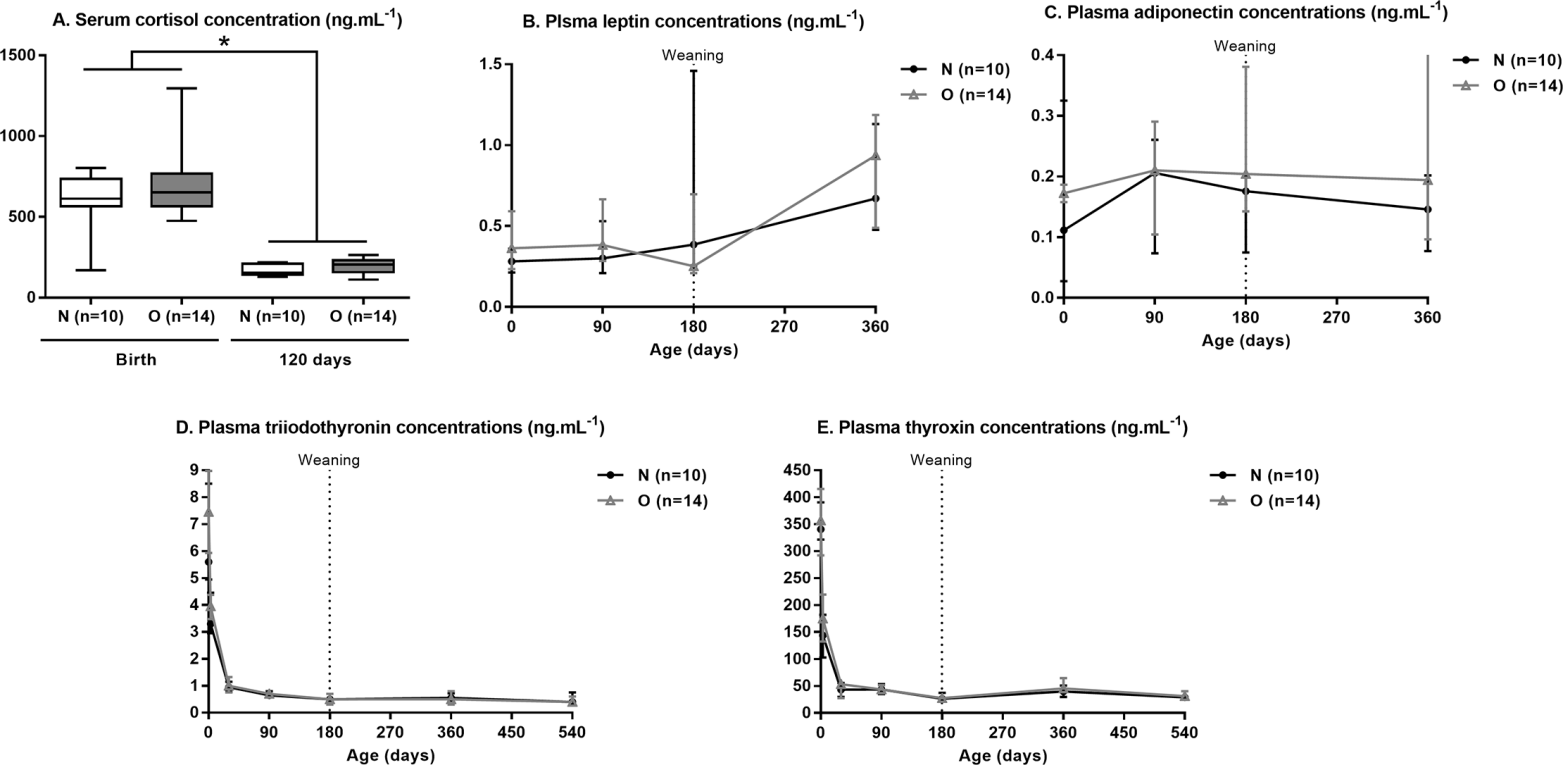
**Serum cortisol (A), plasma leptin (B), adiponectin (C), T3 (D) and T4 (E) concentrations in Normal (n = 10) and Obese (n = 14) foals and yearling between birth and 12 or 18 months of age.** For each time point, asterisks indicate a significant difference between groups (p<0.05).

Plasma concentration of leptin and adiponectin from birth to 12 months of age and that of T3 and T4 from birth to 18 months of age are shown in Fig 9.

Serum concentrations of cortisol at birth and at 4 months did not differ between groups. Serum cortisol concentrations decreased between birth and 4 months of age (p<0.0001).

Plasma leptin and adiponectin concentrations did not differ between groups. Plasma leptin concentrations increased with foal age (p = 0.03), particularly in O foals (p<0.01) but plasma adiponectin did not, without any effect of group:time interaction.

Plasma T3 and T4 concentrations did not differ between groups at any age. Both decreased markedly between birth and 3 months of age (p<0.0001, p<0.0001, respectively) and plateaued thereafter, without any effect of groupe:time interaction.

#### Testes

It was not possible to discriminate between groups using the Principal Component Analysis (S3 Fig).

#### Osteochondrosis

The osteochondrosis status of foals and yearling aged 6, 12 and 18 months is shown in Table 4.

**Table 4:** Number (and percentage) of osteochondrosis (OC) negative and positive Normal (n = 10) and Obese (n = 14) foals and yearlings at 6, 12 and 18 months. The proportion of OC-positive foals and yearlings was not different at 6 and 18 months between groups but there were more OC-positive O yearlings at 12 months compared to N yearlings (p = 0.03).

Age	6 months			12 months			18 months		
	OC-negative	OC-positive	P-value	OC-negative	OC-positive	P-value	OC-negative	OC-positive	P-value
Group “Normal”	7 (70%)	3 (30%)	NS	**9 (90%)**	**1 (10%)**	**0.03**	9 (90%)	1 (10%)	NS
Group “Obese”	9 (64%)	5 (36%)		**6 (47%)**	**7 (53%)**		9 (69%)	4 (30%	

The proportion of foals and yearlings with lesions did not differ between groups at 6 and 18 months of age. At 12 months of age, however, more yearlings of O group (7 over 13) were affected with osteochondrosis lesion than foals of N group (1 over 10, p = 0.03).

## Discussion

In the present study, obese mares were fatter than normal mares from insemination until the end of lactation. Obese mares were more insulin resistant in late gestation, their nitrogen metabolism was altered and they had low-grade inflammation. Moreover, plasma leptin concentrations were increased whereas plasma adiponectin concentration were decreased in obese mares during gestation.

There was no difference in foal birthweight nor subsequent growth between groups. Nevertheless, similar to their dams, foals born to obese mares had an increased low-grade inflammation until 6 months of age compared to controls born to normal dams. They were also more insulin resistant at 6 and 18 months of age. Testicular maturation did not differ between groups, but more foals born to obese mares had osteochondrosis lesions than foals born to normal mares at 12 months of age, although after this age, no differences were detected between the two groups of foals.

### Effect of obesity in pregnant and lactating mares

When they were barren the year before, N mares had a body condition that varied with nutritional offer while O mares had a high, more stable body condition. Again, when pregnant, N mares lost body condition at the end of gestation while O mares maintained their body condition. After foaling, when in pasture, all mares lost body condition during the first months of lactation, but O mares remained fatter. It is important to note that the loss of body condition during lactation occurred at a time of very hot climatic conditions that reduced the nutritional offer to the lactating mares (S1 Text). Thus, despite the drop in BCS during lactation, obese mares can be still considered obese as their BCS was rather stable over the two years and always higher than the body condition of normal mares [11,33].

The increased BCS in the obese group was associated, as expected, with elevated plasma leptin concentrations and decreased plasma adiponectin concentrations in late gestation. These two adipokines are produced by the adipose tissue. In the horse, plasma leptin concentrations are correlated with fat mass while adiponectin concentrations are inversely correlated with fat mass (quantity and BCS) [67–70]. Moreover, it has been shown that insulin resistant obese horses have a higher plasma leptin concentration than insulin sensitive obese horses [35,71]. The observed inverse correlation between plasma leptin concentrations and SI are in agreement with those observations. Similarly, adiponectin is known to improve insulin sensitivity by stimulating adipose tissue and skeletal muscle glucose disposition [72,73] and this is in agreement with the observed positive correlation between plasma adiponectin concentration and SI at 300 days of gestation. Furthermore, adiponectin possesses anti-inflammatory properties and has been shown *in vitro* to decrease cytokine secretion, macrophage pro-inflammatory activity and sensitivity to pro-inflammatory stimuli, as well as to enhance the production of anti-inflammatory cytokines [74–76]. Studies in humans show that plasma adiponectin concentrations are inversely correlated with plasma concentrations of pro-inflammatory cytokines [77,78]. In the non-pregnant horses and ponies, overnutrition with a diet rich in carbohydrates decreases the plasma concentration of adiponectin and significantly increases plasma SAA concentration [34]. Here, obese mares with low plasma adiponectin concentrations had transiently increased plasma SAA concentrations, in accordance with the lower plasma adiponectin concentrations.

In terms of blood biochemistry, plasma NEFA and triglyceride concentrations were not modified in obese mares, in contrast to what is observed in obese humans where increased concentrations of TG and NEFA have been reported [79]. In horses, however, hypertriglyceridemia and increased plasma NEFA concentrations are not always observed in case of obesity [35,80,81].

Obese mares had a decreased concentration of plasma urea nitrogen (BUN) compared to normal mares during gestation. In the rat, obesity due to overnutrition decreases the hepatic production of urea, due to the reduced activity of enzymes involved in the urea cycle [82]. In agreement with these observations, obese growing pigs have shown lower serum urea concentrations than lean pigs [83]. Moreover, plasma amino acid concentrations are modified together with a decrease in hepatic concentrations of gluconeogenic amino acids in prediabetic rats [84]. The decreased plasma urea concentrations observed in obese mares during gestation may be then associated with a shift between amino acid and glucose metabolism in the liver and a decreased hepatic urea production.

Obese mares were more insulin resistant at 300 days of gestation and had a higher glucose effectiveness (Sg, the glucose disposition mediated by insulin independent glucose transporter) compared to N mares. Carter et al., 2010, defined insulin resistance as SI<1.2.10^−4^ L.mUI^-1^.min^-1^, using the 1^st^ and 2^nd^ to lowest reference quintiles for clinically healthy horses [85]. According to this definition, O mares could be considered as insulin resistant. The increase of Sg has been previously observed in obese horses that were insulin resistant as observed in the present study [38]. As insulin resistance was increased in obese horses, the increased Sg may be a compensation to avoid prolonged hyperglycaemia, perhaps due to an increased number or activity of the insulin independent transporters.

In conclusion, obese mares were characterized by an increased fat mass during two consecutive years, although being fed the same quantity and quality of feed as normal mares, altered carbohydrate and nitrogen metabolism as commonly observed in the obese state and increased low-grade inflammation during the second half of gestation.

### Effect of maternal obesity in growing foals and yearlings until 18 months of age

In the present study, we did not observe any difference for growth in foals and yearlings. Only strong alterations of maternal environment such as embryo transfer with breeds differing widely in size, pathology or severe undernutrition have been shown to affect the *in utero* and/or post-natal growth of horses [5]. One study observed a positive correlation between the mean BCS during all pregnancy of the pregnant mare and the birth weight of the foal, but this correlation was very weak (r^2^ = 0.04) [80]. Moreover, foals born to overnourrished mares in gestation that reached obesity at parturition had the same weight at birth than foals born to lean mares [47] but were lighter (-12.2kg, -10%) and smaller (-4cm, -4%) at 2 months of age, possibly due to a decreased milk yield in obese mares [48]. It seems then unlikely that maternal BCS during gestation affects the *in utero* growth of foals. However, Kubiak et al. (1991) were not able to discriminate the effects of maternal overnutrition from the effects of maternal obesity to explain the difference of foal’s growth between birth and 2 months of age [48].

Here, in agreement with the data on growth, there was no difference for thyroid hormone concentrations between groups. In the growing horse, thyroid metabolism has been scarcely described. As observed earlier, thyroid hormones concentrations were elevated at birth and decreased with age [86–90]. In foals, alterations of their concentrations at birth or during growth have been associated with dysmaturity at birth, overgrowth or disease [86,90,91]. Moreover, in horses, hypothyroidism has never been demonstrated to be linked with the equine metabolic syndrome, a pathology defined by regional and or/overall adiposity, insulin resistance, hypertriglyceridemia and hyperleptinemia [41]. Furthermore, thyroidectomized horses do not develop obesity [92].

No difference in testicular maturation were observed between groups. Thyroid hormones have been shown to influence the maturation of Sertoli and Leydig cells in developing testes [93]. IGF-1 has been also shown to stimulate Sertoli cell proliferation in mice [94]. In the growing horse, serum IGF-1 concentrations are correlated with growth rate [95]. In the present study, since we did not observe any difference in growth between groups, we hypothesize then that there was no difference in blood IGF-1 concentrations either.

In line with the absence of differences in growth and thyroid hormones, there were also no differences in plasma leptin nor adiponectin concentrations between groups. In agreement with these observations, foals born to hyperleptinemic mares did not have elevated plasma leptin concentration from birth until 4 days of age [96]. These observations do not exclude a transient elevation of plasma leptin concentrations. Indeed, others observed that foals born to hyperleptinemic dams have higher plasma leptin concentrations from 6 to 18 hours after birth. In contrast, no difference was observed at birth and 24 hours after birth compared to control foals [97].

In contrast to the previously discussed lack of differences, O foals had increased plasma SAA concentration compared to N foals until 6 months of age. This observation may be due to differences in the composition of maternal milk as it disappeared at 12 months of age. The increased SAA concentration in O foals until 6 months of age reflects an increased systemic inflammation. In humans and rats, maternal low-grade inflammation associated with obesity is transferred to the offspring, in particular in the brain, leading to behaviour changes such as increased anxiety, increased behavioural abnormalities and reduced spatial learning abilities [98,99]. In the present study, it could then be hypothesized that the alterations of maternal phenotype could affect foal behaviour. In this species, decreased learning abilities and increased behavioural problems could possibly affect performance in sport and increase the incidence of dangerous behaviours.

Finally, we observed a link between maternal and post-natal environment on foal development. At 6 and 18 months of age, after being in pasture and/or suckling their dams, O foals were more insulin resistant than N foals without a difference in terms of osteochondrosis lesions. In contrast, at 12 months of age, after a winter indoors during which they were fed twice a day with concentrates, N yearlings were more metabolically affected by carbohydrate rich meals than O yearlings because their insulin sensitivity decreased between 6 and 12 months of age while the insulin sensitivity of O yearlings was not affected by feeding. This observation is in line with data from a previous study comparing metabolic responses of overfeeding between foals that were insulin resistant at 6 months of age compared to controls [51]. The absence of statistically significant difference for osteochondrosis lesions between groups at 18 months, however, needs to be confirmed as the statistical power of the chi-squared test was very low (22%). As a matter of fact, > 30% of the O foals had lesions compared to 10% of the N foals.

Osteochondrosis is a dynamic, multifactorial disease. Epidemiological and experimental studies have linked osteochondrosis lesions in foals with feeding of pregnant mares and growing foals with cereals [59,100,101]. Moreover, foals that are most affected by osteochondrosis have increased post-prandial blood glucose and insulin compared to non-affected foals [102,103]. *In vitro*, insulin stimulates the differentiation of pre-osteoblasts into osteoblasts [104–107], as well as chondrocyte survival and proliferation [108,109] and cartilaginous growth [110]. Which one of hyperinsulinemia or insulin resistance in chondrocytes is responsible for the defects in cartilage maturation remains unknown. In the present study, post-prandial plasma glucose and insulin concentrations were not analysed. Low-grade inflammation may also play a role as TNF-α and IL-6 are known to inhibit bone growth [111–114]. Moreover, a functional analysis has shown that leukocytes of horses affected by osteochondrosis presented alterations of genes involved in insulin metabolism and inflammation [115]. More work is needed to understand better the role of energy metabolism and low-grade inflammation in cartilage maturation and development of transient osteochondrosis lesions.

In conclusion, maternal obesity was associated with enhanced insulin resistance, low-grade inflammation and the transient development of osteochondrosis lesions in foals. These may affect both physical and behavioural performances at adulthood, with particular importance in a species dedicated to sport and competition. Maternal obesity during gestation should then be avoided to protect the foals from its deleterious effects. Since we and others previously demonstrated that undernutrition during pregnancy also affects the development of foals, at least until 24 months of age [51,100,116–118], it is important to underline that maternal undernutrition should also be avoided.

### Main limitation

The main limitation of this study is the potential interaction of genetics and environment on foal development.

In the present study, we tried to smooth out the effects of genetics by deciding to use the semen of only one stallion to produce the foals in order to remove the paternal effect. The genealogy study of mares demonstrated that genetic backgrounds were shared in the two groups, with an important number of stallions used to produce broodmares. Moreover, the pregnant mares belonged to the sport breeds “French Anglo-Arab” and “Selle Français”, that are not known to be prone to develop obesity nor to being insulin resistant (in contrast to ponies) [11,119,120]. Pregnant mares’ obesity is thus most probably due to their environmental breeding conditions before the beginning of the study and not solely the effect of genetics.

## Conclusion

In the present study, we showed that maternal obesity, associated with insulin resistance, alters the secretion of adipokines and increases low-grade inflammation in pregnant mares. These alterations of maternal environment increase the low-grade inflammation, the insulin resistance and the transient development of osteochondrosis lesions in foals and yearlings until 18 months of age.

In conclusion, gestational obesity could possibly induce insidious consequences on health, well-being and athletic performances of adult horses and subsequently putatively affect the whole equine industry.

The main recommendation arising from this work is that broodmares should be maintained at an optimal body condition (around 3/5 or 6/9 depending on the scale) during pregnancy.

## Supporting information

S1 Table Sire, dam and sire of dam of the different broodmares of Obese or Normal groups used in the study. Sires, dams and grand sires that produced several broodmares are highlighted in colours.
(DOCX)

S2 Table Nutritional information of pregnant mares. A. Daily nutritional supply ingested by pregnant mares during wintering (from 6 months of gestation to foaling) (median and [Q1-Q3]). B. Quality of feedstuff from the INRA system given to pregnant mares during wintering (from 6 months of gestation to foaling). HFU: Horse feed unit (net energy, 1 HFU = 2250 kcal), HDCP: Horse digestible crude protein, RC: Raw cellulose, P: Phosphorus, Ca: Calcium.
(DOCX)

S3 Table Nutritional information of growing foals. A. Daily nutritional supply ingested by growing foals during wintering (from 6 to 12 months of age) (median and [Q1-Q3]). B. Quality of feedstuff from the INRA system given to pregnant mares during wintering (from 6 to 12 months of age). HFU: Horse feed unit (net energy, 1 HFU = 2250 kcal), HDCP: Horse digestible crude protein, RC: Raw cellulose, P: Phosphorus, Ca: Calcium.
(DOCX)

S4 Table Composition of the vitamin and minerals complement given to foals and yearlings in pasture. (DOCX)

S1 Fig Validations of leptin assay. A. Dilutional parallelism between standard curve and endogenous leptin. To linearize the 4PL curve, logit was calculated as logit = log ((AlphaLISA signal (count)–minimum asymptote) / (maximum asymptote–AlphaLISA signal (count))). B. Expected leptin values against obtained values. Linear regression statistic test was applied to compare the equality of slope to 1 and intercepts to 0. Run-test was performed to determine whether data deviated significantly from the linear model. For both tests and for all samples, p<0.5. C. Bland-Altman graph comparing the expected leptin values against the obtained values.
(TIF)

S2 Fig Validations of adiponectin assay. A. Dilutional parallelism between standard curve and endogenous adiponectin. To linearize the 4PL curve, logit was calculated as logit = log ((AlphaLISA signal (count)–minimum asymptote) / (maximum asymptote–AlphaLISA signal (count))). B. Expected adiponectin values against obtained values. Linear regression statistic test was applied to compare the equality of slope to 1 and intercepts to 0. Run-test was performed to determine whether data deviated significantly from the linear model. For both tests and for all samples, p<0.5. C. Bland-Altman graph comparing the expected adiponectin values against the obtained values.
(TIF)

S3 Fig Principal component analysis of expression of genes involved in testicular maturity (*Cyp19*, *Connexin 43* (*Cx43*), *Androgen Receptor* (*AR*), *Steroidogenic Acute Regulatory Protein* (*STAR*)), testicular immaturity (*Anti-Müllerian hormone* (*AMH*)) and genes involved in the establishment of the blood-testis barrier (*Occludin and N Cadherin*). A. Graph of variables. B. Graph of individuals. There was no difference between groups.
(TIF)

S1 Text Weather report edited by Météo-France for the months of July and August when broodmares were in lactation in pastures. (PDF)
