# Supplementary material for: Maternal obesity increases insulin resistance, low-grade inflammation and osteochondrosis lesions in foals and yearlings until 18 months of age
Source: PLoS One. 2018 Jan 26;13(1):e0190309. doi: 10.1371/journal.pone.0190309 (PMC5786290; doi:10.1371/journal.pone.0190309)
Supplement: S1 Table — Sires, dams and grand sires that produced several broodmares are highlighted in colours. (DOCX) [file pone.0190309.s001.docx]

| **Mare** | **Group** | **Sire** | **Dam** | **Sire of dam** |
| --- | --- | --- | --- | --- |
| 1 | Obese | Allegro Fontaine | On the road | Daguet de terlong |
| 2 | Obese | Allegro Fontaine | Gipsy queen du roc | Donald duck |
| 3 | Obese | Allegro Fontaine | Melisande | Quinsac |
| 4 | Obese | Dollar du Mesnil | Farisette | Dan music |
| 5 | Obese | El Paso Platière | Austin | Mad captain |
| 6 | Obese | El Paso Platière | Etila | Tams du plessis |
| 7 | Obese | Florian du Paon | Brenda Louvo | King's road |
| 8 | Obese | Florian du Paon | Api Maison Rouge | Nobel |
| 9 | Obese | Hurlevent de Breka | Here | Kesberoy |
| 10 | Obese | Hurlevent de Breka | Line Breeze | Phantom breeze |
| 11 | Obese | Must'pom | Megere | Bonaparte du hillo |
| 12 | Obese | Must'pom | K'eseksa | Quinsac |
| 13 | Obese | No Comment Chayottes | Cellia de Fongibe | Black beauty ii |
| 14 | Obese | Type d'Elle | Baselle II | Qolt blond |
| 15 | Normal | Allegro Fontaine | Indiana Jolie | Xenophon |
| 16 | Normal | Belo des vernieres | Loucry | Stay for lunch |
| 17 | Normal | Brandy de la Cour | Line Breeze | Phantom breeze |
| 18 | Normal | Dollar du Mesnil | Austin | Mad captain |
| 19 | Normal | Must'pom | La Valade | Uragan |
| 20 | Normal | Must'pom | Vobiscum | What a joy |
| 21 | Normal | No Comment Chayottes | Qaldeenne | Frou frou |
| 22 | Normal | No Comment Chayottes | Lenticule | Quinsac |
| 23 | Normal | No Comment Chayottes | Idole de la cour | Shaliman du thot |
| 24 | Normal | Tcherkou | Belle Fille | Dhaudevi |
