# Supplementary material for: Maternal obesity increases insulin resistance, low-grade inflammation and osteochondrosis lesions in foals and yearlings until 18 months of age
Source: PLoS One. 2018 Jan 26;13(1):e0190309. doi: 10.1371/journal.pone.0190309 (PMC5786290; doi:10.1371/journal.pone.0190309)
Supplement: S2 Table — A. Daily nutritional supply ingested by pregnant mares during wintering (from 6 months of gestation to foaling) (median and [Q1-Q3]). B. Quality of feedstuff from the INRA system given to pregnant mares during wintering (from 6 months of gestation to foaling). HFU: Horse feed unit (net energy, 1 HFU = 2250 kcal), HDCP: Horse digestible crude protein, RC: Raw cellulose, P: Phosphorus, Ca: Calcium. (DOCX) [file pone.0190309.s002.docx]

| **A** | **Hay (kg of dry matter)** | | | | | **Haylage (kg of dry matter)** | | | | | **Concentrate (kg of dry matter)** | | | | |
| --- | --- | --- | --- | --- | --- | --- | --- | --- | --- | --- | --- | --- | --- | --- | --- |
| Month of gestation | 170 | 174 | 240 | 270 | 300 | 180 | 210 | 240 | 270 | 300 | 180 | 210 | 240 | 270 | 300 |
| Group Normal (n=10) | 5.3  [5.2-5.3] | 5.3  [5.2-5.3] | 5.2  [5.0-5.3] | 5.3  [5.3-5.6] | 5.8  [5.6-6.0] | 2.8  [2.8-2.8] | 3.6  [3.6-3.6] | 3.9  [3.9-3.9] | 4.1  [4.1-4.1] | 4.3  [4.3-4.3] | 2.1  [2.1-2.1] | 2.1  [2.1-2.1] | 2.4  [2.4-2.4] | 2.6  [2.6-2.6] | 2.6  [2.6-2.6] |
| Group Obese (n=14) | 5.3  [5.2-5.3] | 5.3  5.2-5.3] | 5.2  [5.1-5.2] | 5.6  [5.5-5.7] | 5.9  [5.7-6.1] | 2.8  [2.8-2.8] | 3.6  [3.6-3.6] | 3.9  [3.9-3.9] | 4.0  [4.0-4.0] | 4.2  [4.2-4.2] | 2.6  [2.6-2.6] | 2.6  [2.6-2.6] | 2.9  [2.9-2.9] | 3.0  [3.0-3.0] | 3.0  [3.0-3.0] |

| **B** | **Hay** | | | | | **Haylage 1 (used until the 30^th^ of April)** | | | | | **Haylage 2 (Used from the 1^st^ of May)** | | | | | **Concentrate** | | | | |
| --- | --- | --- | --- | --- | --- | --- | --- | --- | --- | --- | --- | --- | --- | --- | --- | --- | --- | --- | --- | --- |
|  | HFU | HDCP (g) | RC (g) | P (g) | Ca (g) | HFU | HDCP (g) | RC (g) | P (g) | Ca (g) | HFU | HDCP (g) | RC (g) | P (g) | Ca (g) | HFU | HDCP (g) | RC (g) | P (g) | Ca (g) |
|  | 0.86 | 72.0 | 364.3 | 1.7 | 3.2 | 0.6 | 44.0 | 276.0 | 3.4 | 4.9 | 0.6 | 45.6 | 302.6 | 0.9 | 1.5 | 1.1 | 93.5 | 53.0 | 5.3 | 11.0 |
