# Supplementary material for: Maternal obesity increases insulin resistance, low-grade inflammation and osteochondrosis lesions in foals and yearlings until 18 months of age
Source: PLoS One. 2018 Jan 26;13(1):e0190309. doi: 10.1371/journal.pone.0190309 (PMC5786290; doi:10.1371/journal.pone.0190309)
Supplement: S3 Table — A. Daily nutritional supply ingested by growing foals during wintering (from 6 to 12 months of age) (median and [Q1-Q3]). B. Quality of feedstuff from the INRA system given to pregnant mares during wintering (from 6 to 12 months of age). HFU: Horse feed unit (net energy, 1 HFU = 2250 kcal), HDCP: Horse digestible crude protein, RC: Raw cellulose, P: Phosphorus, Ca: Calcium. (DOCX) [file pone.0190309.s003.docx]

| **A** | **Hay (kg of dry matter)** | **Concentrate (kg of dry matter)** |
| --- | --- | --- |
| Until 11 months of age | 5.0 | 1.6 |
| Between 11 and 12 months of age | 4.3 | 2.7 |

| **B** | **Hay** | | | | | **Concentrate** | | | | |
| --- | --- | --- | --- | --- | --- | --- | --- | --- | --- | --- |
|  | HFU | HDCP (g) | RC (g) | P (g) | Ca (g) | HFU | HDCP (g) | RC (g) | P (g) | Ca (g) |
|  | 0.6 | 72 | 364.3 | 1.73 | 3.2 | 1.1 | 102 | 63.9 | 4.9 | 7.1 |
