# Supplementary figures and images for: Maternal obesity increases insulin resistance, low-grade inflammation and osteochondrosis lesions in foals and yearlings until 18 months of age
Source: PLoS One. 2018 Jan 26;13(1):e0190309. doi: 10.1371/journal.pone.0190309 (PMC5786290; doi:10.1371/journal.pone.0190309)

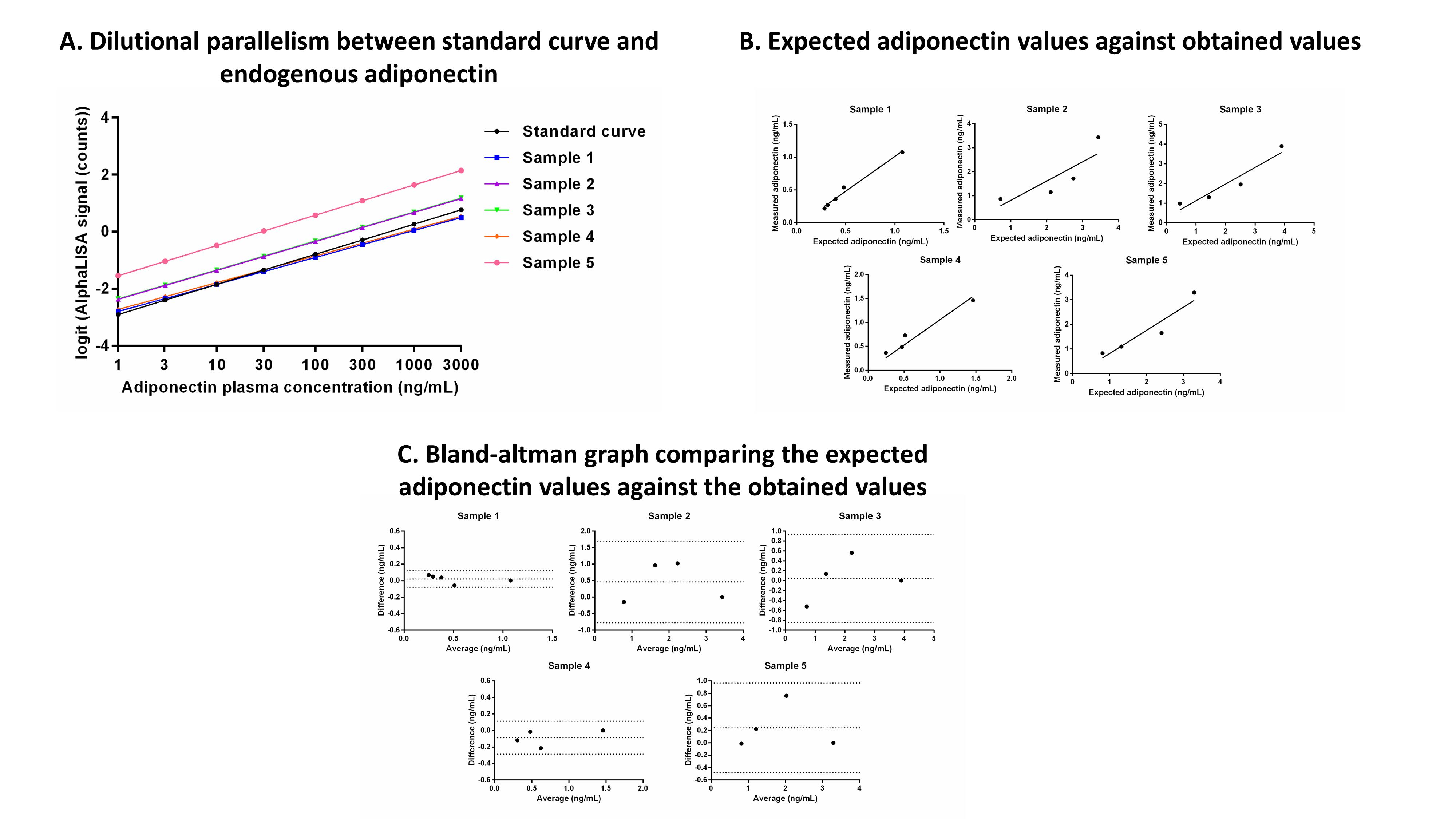

Supplement: S1 Fig — A. Dilutional parallelism between standard curve and endogenous leptin. To linearize the 4PL curve, logit was calculated as logit = log ((AlphaLISA signal (count)–minimum asymptote) / (maximum asymptote–AlphaLISA signal (count))). B. Expected leptin values against obtained values. Linear regression statistic test was applied to compare the equality of slope to 1 and intercepts to 0. Run-test was performed to determine whether data deviated significantly from the linear model. For both tests and for all samples, p<0.5. C. Bland-Altman graph comparing the expected leptin values against the obtained values. (TIF) [file pone.0190309.s005.tif]

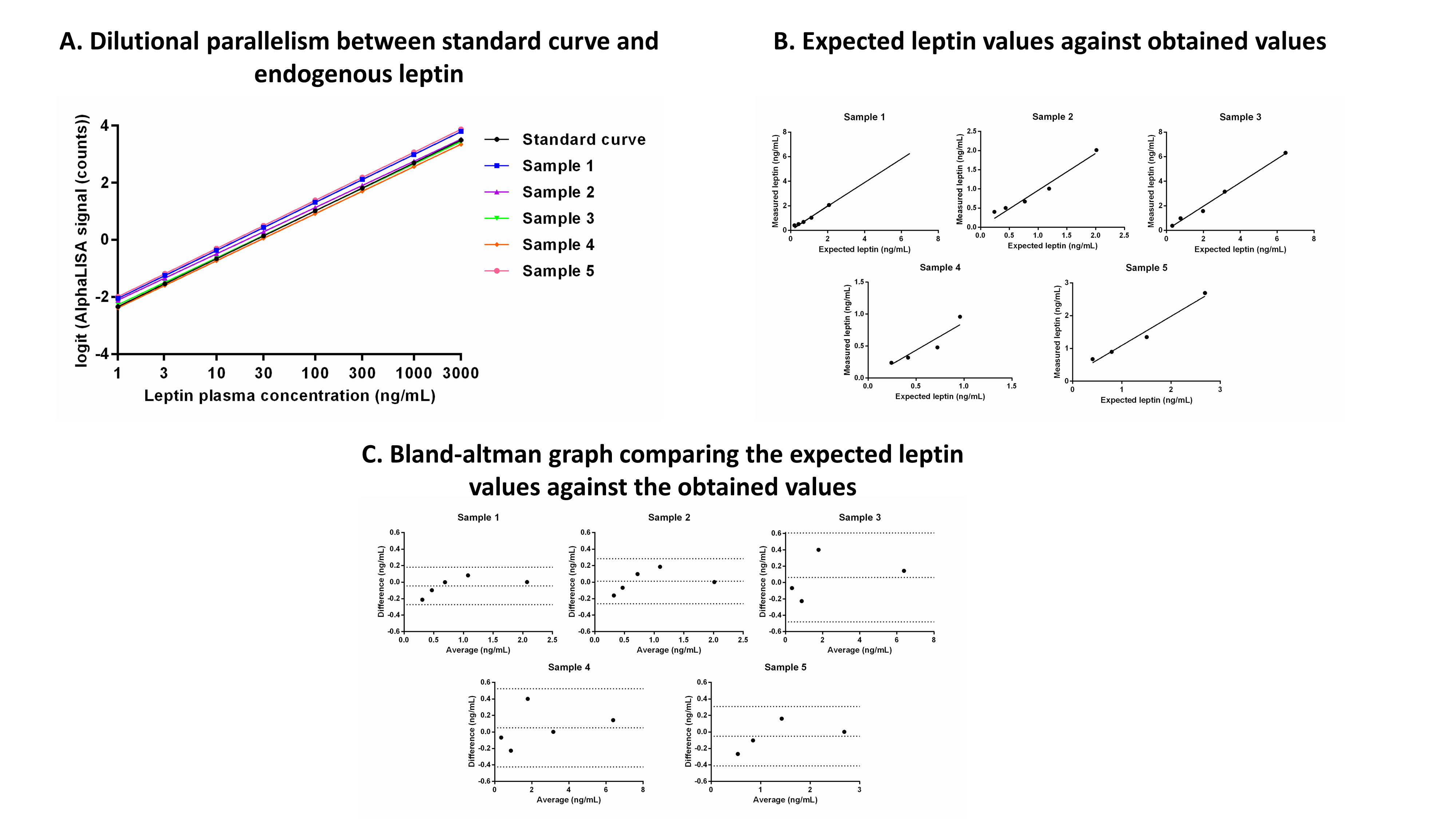

Supplement: S2 Fig — A. Dilutional parallelism between standard curve and endogenous adiponectin. To linearize the 4PL curve, logit was calculated as logit = log ((AlphaLISA signal (count)–minimum asymptote) / (maximum asymptote–AlphaLISA signal (count))). B. Expected adiponectin values against obtained values. Linear regression statistic test was applied to compare the equality of slope to 1 and intercepts to 0. Run-test was performed to determine whether data deviated significantly from the linear model. For both tests and for all samples, p<0.5. C. Bland-Altman graph comparing the expected adiponectin values against the obtained values. (TIF) [file pone.0190309.s006.tif]

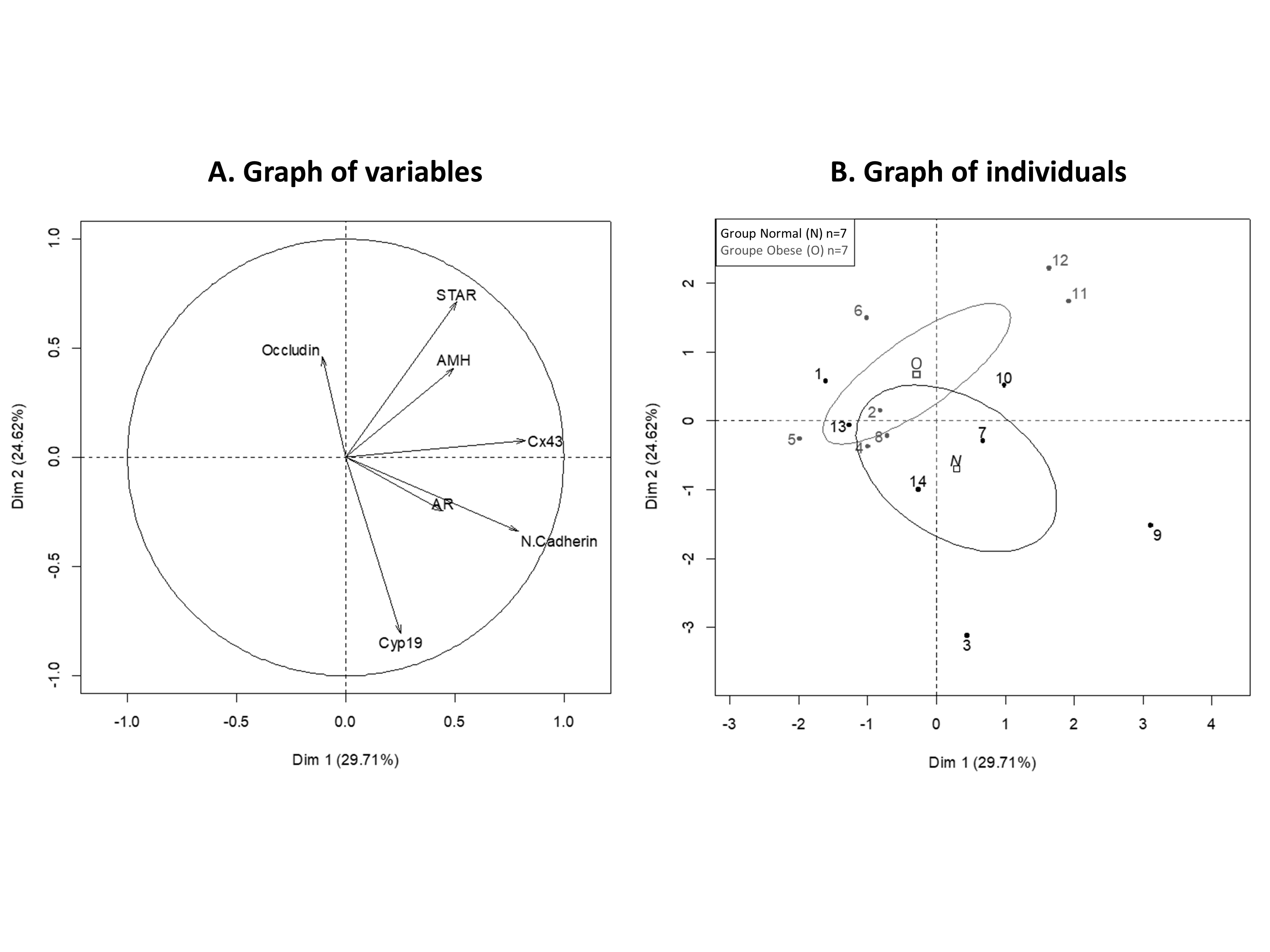

Supplement: S3 Fig — A. Graph of variables. B. Graph of individuals. There was no difference between groups. (TIF) [file pone.0190309.s007.tif]
