# Supplementary material for: Maternal obesity increases insulin resistance, low-grade inflammation and osteochondrosis lesions in foals and yearlings until 18 months of age
Source: PLoS One. 2018 Jan 26;13(1):e0190309. doi: 10.1371/journal.pone.0190309 (PMC5786290; doi:10.1371/journal.pone.0190309)
Supplement: S1 Text — (PDF) [file pone.0190309.s008.pdf]

Bulletin climatique

Limousin

Juillet 2015

Exceptionnellement sec, très chaud

Précipitations mensuelles (mm)

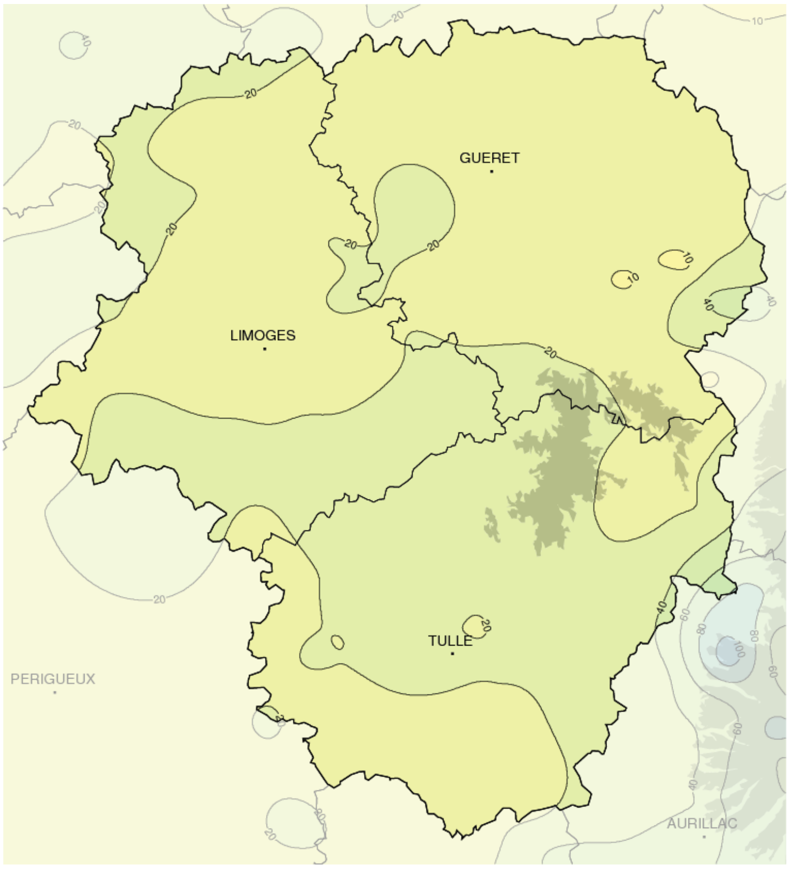

Caractère dominant du mois
Il est très chaud, sec et bien ensoleillé

Précipitations
Elles sont très faibles et déficitaires sur l'ensemble de la région, ce déficit atteignant 50 à 88% par rapport aux normales.

Températures
Les moyennes sont chaudes, supérieures aux normales de 2 à 4°C, avec une première partie de mois très chaude voire caniculaire par moments.

Ensoleillement
Il est assez généreux et supérieur là aussi aux normales de 12 à 20%.

Faits marquants

Sécheresse remarquable

Ce mois de Juillet est très sec . Il se classe au 1er rang des mois de Juillet les plus secs depuis 1973 à Limoges Bellegarde et à Brive depuis 1988. A Guéret ce mois de Juillet est au 3ème rang depuis 1976, avec seulement 2 mm de plus que le mois le plus sec. En outre, le nombre de jours avec précipitations supérieures à 1 mm est de seulement 4 alors que la normale est de 9.
Ce temps sec vient aggraver fortement l'état de sécheresse amorcé depuis le mois d'avril en Limousin.

Un 16 Juillet record

Le 16 Juillet restera dans les annales. En effet de nombreux records absolus de température maximale ont été battus ce jour là. C'est le cas à Brive avec 41.4°C, à Limoges ville avec 40°C, à Boussac (23) avec 38.9°C entre autres pour ne citer que les valeurs les plus hautes de chaque département. Il faut également noter le nombre de jours très chauds (maximales supérieures à 30°C) enregistré, qui est de 9 à Limoges alors que la normale pour un mois de Juillet est seulement de 3 jours.

METEO FRANCE
Toujours un temps d'avance

Précipitations

Très sec

Les précipitations sont très nettement déficitaires, il s'agit quasiment partout du mois de Juillet le plus sec de ces trente dernières années. Le mois commence avec des pluies très faibles du 1er au 4 (de 1 à 4 mm en cumul) puis après un léger passage pluvieux le 7 (0 à 3 mm), il faut attendre le 16 pour voir quelques averses orageuses se produire en Corrèze. Le temps est à nouveau instable le 18, mais seuls la Corrèze et le Sud-Est de la Creuse sont touchés. Les 21 et 22 des averses se produisent à nouveau de façon isolée donnant jusqu'à 13 mm à Bénévent(23). Du 24 au 27, il pleut très faiblement chaque jour, puis les 28 et 29 les pluies sont plus marquées (jusqu'à 17 mm à Surdoux(87)). Le 31 il pleut à nouveau faiblement.

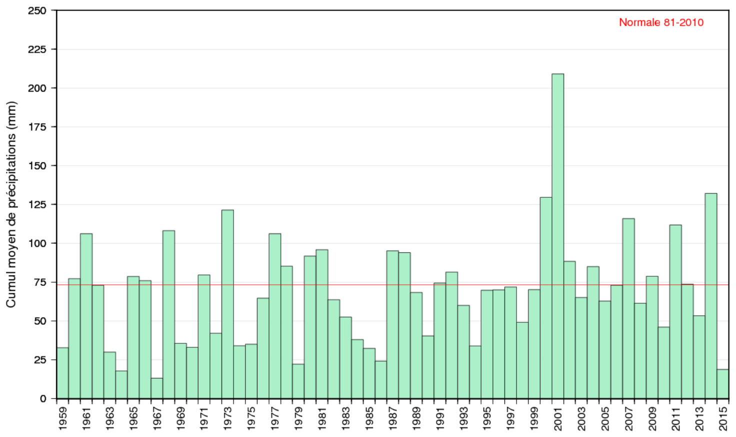

Cumul mensuel de précipitations depuis 1959 sur la région Limousin

Ensoleillement

Soleil généreux

Le soleil est bien présent mais pas autant que le laissent à penser les températures élevées. Il brille durant 305 h à Brive (+19% par rapport à la normale) et 269 h à Limoges (+13% par rapport à la normale). L'ensoleillement est de 277 h à Naves (19), 285 h à Saint-Yrieix(87) et 286 h à Guéret et Bourganeuf(23).

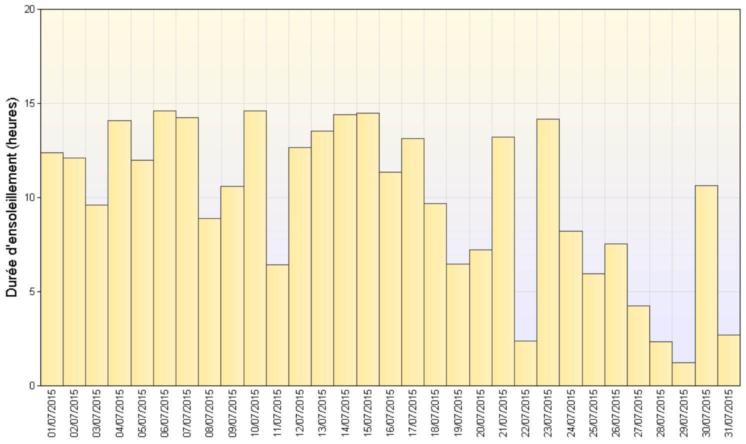

Ensoleillement quotidien à Brive (19)

Vents

Sud-Ouest dominant

Le vent est faible à modéré de Sud-Ouest à Ouest dominant. Il s'oriente toutefois par moments au Nord-Est.

Rafales maximales enregistrées:

Le 19:
Ussel (19) 61 km/h
Nespouls (19) 60 km/h

Le 24
Peyrelevade (19) 60 km/h
Magnac-Laval (87) 59 km/h

Le 26
Felletin (23) 58 km/h

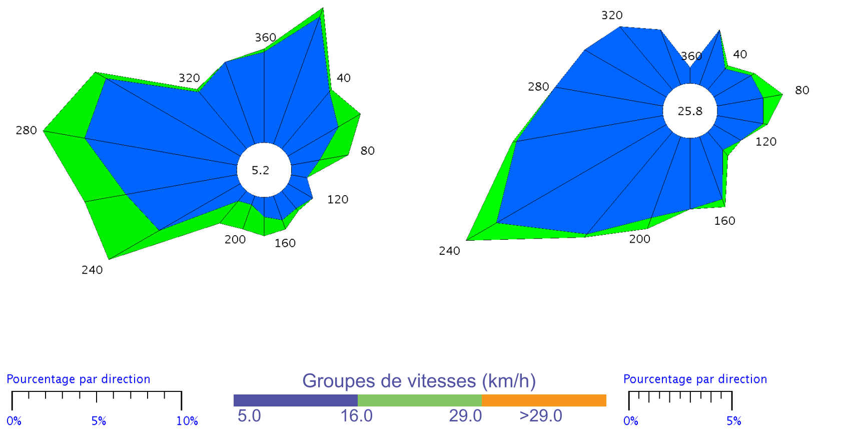

Limoges (87) *La Souterraine (23)*

Rose des vents

METEO FRANCE
Toujours un temps d'avance

Températures

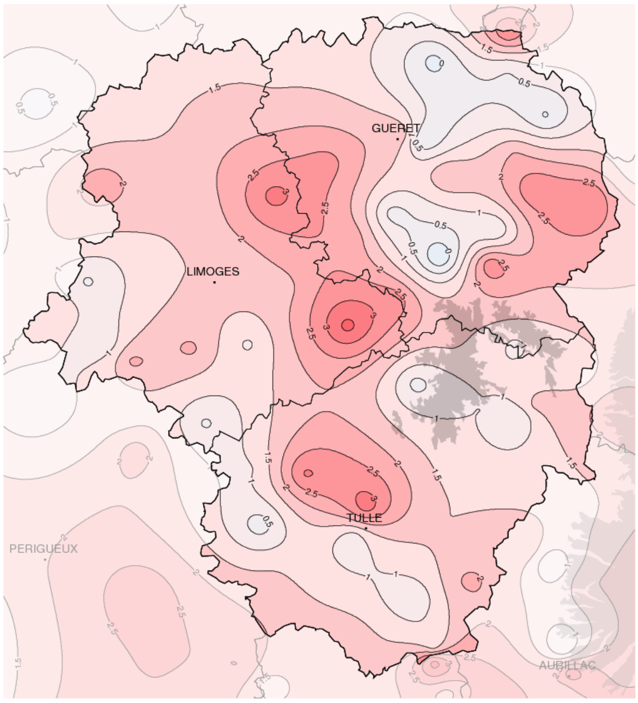

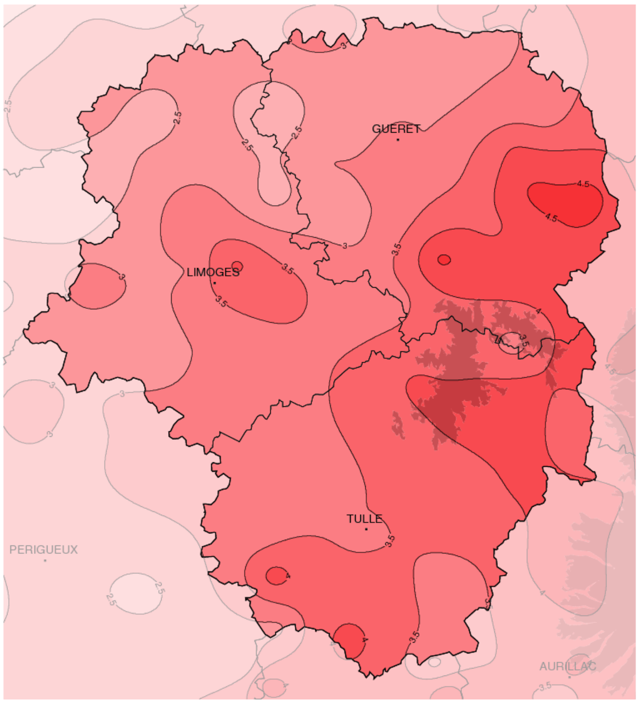

Températures minimales (°C)

Températures maximales (°C)

Ecart à la normale mensuelle (*)

Première quinzaine très chaude

La canicule démarrée fin Juin se poursuit début Juillet avec des minimales et des maximales nettement au-dessus des normales. Localement le 3 les maximales battent des records, elles atteignent ou dépassent les 33°C sur l'ensemble du Limousin. Il fait même 40°C à Brive, 39°C à Argentat(19), 38°C à Limoges ville, Saint-Loup et Auzances(23). A partir du 4 les températures nocturnes fléchissent légèrement mais les températures maximales restent élevées. Elles sont moins fortes toutefois avec un passage en-dessous des normales le 8. Le 10 nous connaissons un nouveau pic de chaleur avec 30 à 37°C sur la région. Les jours suivant il fait moins chaud puis les 15 et 16 les maximales repartent à la hausse. Elles atteignent leur apogée le 16 où les records absolus de températures sont battus quasiment sur l'ensemble de la région. Il fait 41°C à Brive, Argentat et Branceilles(19) et 40°C à Limoges ville et Saint-Loup et plus de 35°C sur tout le Limousin. La chaleur est moins marquée les jours suivant malgré une nouvelle poussée du mercure le 21. A partir du 22, les températures baissent et repassent en-dessous des normales. La journée la plus fraîche est le 29 avec des maximales ne dépassant pas les 23°C. Le lendemain matin l'atmosphère est très fraiche, avec seulement 2 à 3°C sur le Millevaches. Le mois se termine avec des températures qui restent en-dessous des normales.

Ecart à la normale mensuelle (*) des températures moyennes mensuelles pour les 30 dernières années à Limoges (87)

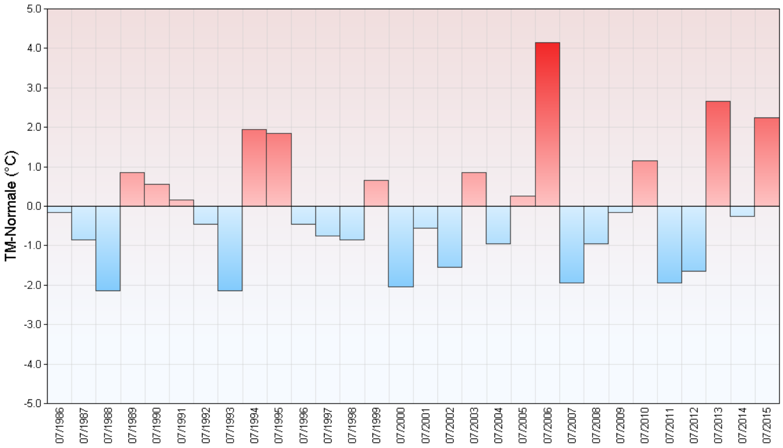

(*) Période de référence 1981/2010

METEO FRANCE
Toujours un temps d'avance

Résumé mensuel

Du 1er au 24: Très chaud avec de rares orages

Le mois débute dans un flux de Sud et une chaleur étouffante et même des températures caniculaires du 1er au 3 sur Creuse et Haute-Vienne et jusqu'au 4 sur la Corrèze. Le 3 les températures maximales flirtent avec les 40°C dans le Sud de la Corrèze avec 39.6°C à Brive et 39°C Argentat. Le 4, il fait très chaud le matin puis en marge d'une perturbation quelques averses orageuses se produisent. Le 5, à l'arrière des averses orageuses le temps est un peu moins chaud mais ensoleillé; les 6 et 7 le mercure dépasse à nouveau 30°C sur l'ensemble de la région. Après un passage faiblement perturbé dans la nuit, le 8 le temps est plus frais. Les 9 et 10 voient l'atmosphère se réchauffer sous un ciel de moins en moins nuageux. Le 11 en début de journée le ciel couvert laisse échapper de très faibles bruines, mais en cours de journée le ciel se dégage. Du 12 au 14 Juillet il fait moins chaud sous un ciel voilé à couvert. Le ciel se dégage le 15 et le mercure remonte, le 16 est caniculaire sous un soleil de plomb avec des maximales record et quelques averses orageuses. Les 17 et 18 restent très chauds et encore localement instables avec des orages plus marqués le 18 sur le Sud de la région. Le temps devient moins chaud les jours suivants et du 19 au 24 des averses orageuses isolées se produisent par endroits.

Du 25 au 31: Plus frais et faiblement pluvieux

Un régime d'Ouest modéré se met en place dans une atmosphère rafraichie. Les 26 et 27, sous un ciel très nuageux des averses se produisent. Les 28 et 29, une perturbation traverse la région donnant enfin de la pluie pour tous. Après une accalmie le 30, des pluies faibles se produisent à nouveau le 31.

Brive (19)

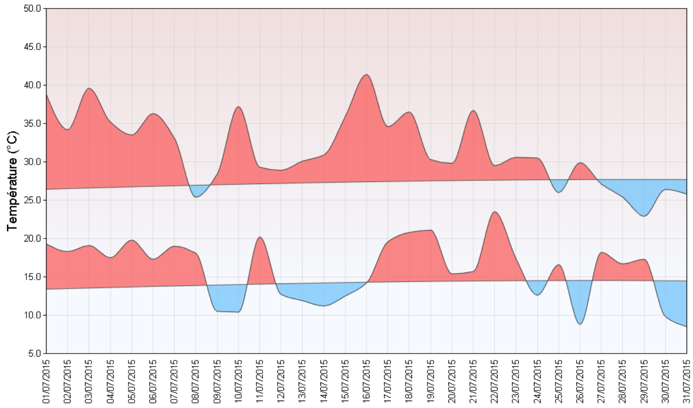

Tulle (19)

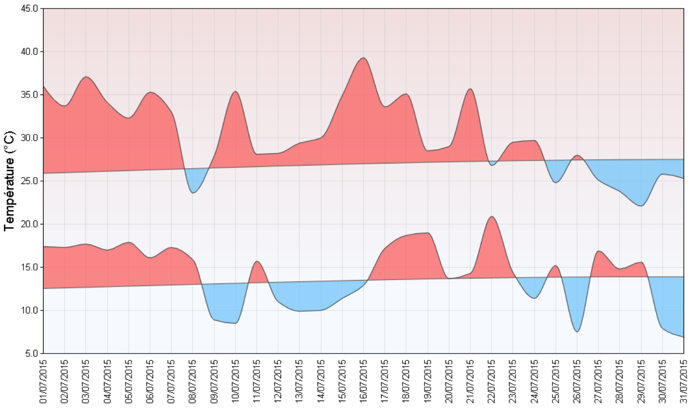

Températures minimales et maximales quotidiennes

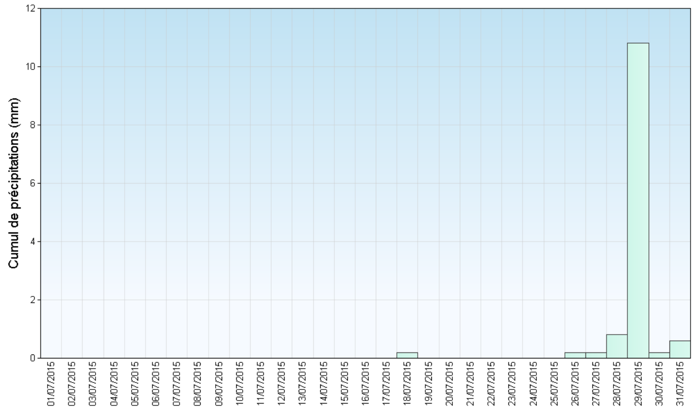

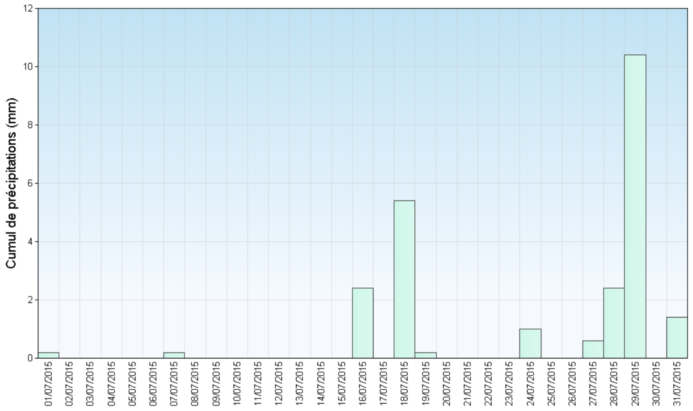

Précipitations quotidiennes

Directeur de publication : Jean-Marc LACAVE
Rédaction : CM-LIMOGES

METEO FRANCE
Toujours un temps d'avance
